# Numerical investigation of steel-concrete composite (SCC) beam subjected to combined blast-impact loading

**DOI:** 10.1016/j.heliyon.2022.e10672

**Published:** 2022-09-17

**Authors:** Tesfaye Alemu Mohammed, Solomon Abebe

**Affiliations:** a Construction Quality and Technology Center of Excellence, Addis Ababa Science and Technology University, Addis Ababa, Ethiopia; b Department of Civil Engineering, Addis Ababa Science and Technology University, Addis Ababa, Ethiopia; c Department of Civil Engineering, Debre Markos University, Debre Markos, Ethiopia

**Keywords:** Blast load, Finite element analysis, Impact load, Steel-concrete composite beam

## Abstract

Existing literatures on combined effects of blast-impact loadings on steel-concrete composite beams are limited. In this study, behavior of steel-concrete composite beam subjected to combined blast-impact loading was investigated using LS-DYNA nonlinear FEA software program. The accuracy and reliability of developed FEA model was validated using experimental data reported in literature. Parametric studies were performed on impacting speed, concrete strength, various characteristics of H-structural steel, yield strength of studs and steel rebars to get insight into performance of composite beam subjected to combined blast impact loading. FEA results revealed that increase in specified cylindrical compressive strength of concrete, yield stress, flange and web thicknesses of H-type structural steel beam significantly improved dynamic response of a steel-concrete composite beam under combined impact-blast load case whereas yield stress of studs, and reinforcement steel bars showed insignificant contribution. Moreover, increasing impactor’s initial velocity significantly affects dynamic response of a SCC beam under combined blast-impact loading.

## 1 Introduction

An explosion occurs when there is a spontaneous expansion and reaction of matters. This sudden release in energy is then accompanied by four effects namely: high temperature, sound, light and blast wave. Among the four explosive effects, blast wave first reaches and strikes a structure and apply a shock front pressure which originally expands outward from the surface of explosive into surrounding air in radial formations [1]. Easy production, delivering, and detonation systems of explosives makes a suitable and comfortable platform for extremists to prefer explosion attacks for their strategies. Furthermore, limited accessibility and confidentiality of manuals makes blast-effect studies more difficult. Most of imperative manuals and computer codes are accessible for military sectors and for design of their associated reinforced concrete facilities such as bunkers, domed shelters, ammunition centers, command and control centers. As a result, most of the available information on the blast science is on reinforced concrete structures [2]. As a result, most of the available information on the blast resistant design focused reinforced concrete structures, and further extended studies and investigations on a steel concrete composite beams is required Astaneh-Asl [2]. After arrival of blast induced shock waves, secondary loading including fragments and debris hit a structure revealing an impactive force effects. Thus, further studies and information on steel and composite structural members subjected to combined blast-impact loadings is crucial.

A composite member is consisting of concrete and structural or cold-formed steel, interconnected by studs so as to limit longitudinal slip between concrete and steel and thus separation of one component from the other Eurocode-4 [3]. The use of steel-concrete composite (SCC) which are normally hot rolled or fabricated steel sections that act compositely with reinforced concrete slab makes it efficient for long and high-rise buildings and bridges. The main advantage of composite usage is concrete’s efficiency in compression and steels in tension; concrete encasement restraints steel against buckling; steel provides good ductility and concrete brings protection against corrosion and fire. The required composite interaction and bond is achieved by use of studs to connect top flange of a structural steel into concrete beam. This composite action increases load carrying capacity and stiffness of a composite beam by factors ranging from 2 to 3.5 [4].

Astaneh-Asl [2] reported one of solutions to problems associated with structural steel such as undesirable failure modes including local buckling, lateral torsional buckling and distortion of steel cross-sections (flange folding and web dishing deformations) under explosions can be compensated by using composite structures. Therefore, this research investigates application of composite members to alleviate aforementioned undesirable failure modes under combined blast impact loading. Eurocode-4 [3] defines steel-concrete composite (SCC) beam as a composite member subjected primarily to bending. This composite structural member has different constitutive elements including concrete slab, reinforcement bars, studs, and structural steel beam however there is a need to investigate composite behavior and nonlinear response of those elements in combined blast-impact loading conditions.

Zhang et al. [5] numerically evaluated nonlinear dynamic response of RC beams when subjected to combined blast and impact loads using LS-DYNA. The authors considered effect of different beam depths, span lengths, reinforcement configurations, and impacting loading on blast response on a RC beam. The nonlinear analysis on RC beam under combined effects revealed spalling and global flexural damages increased with decreasing beam depth and increasing span length. Other researchers [6, 7, 8, 9] have studied a dynamic response of SCC beams based on varying applied loading system. Considered loading systems includes: monotonic, shearing, and vertical point loads. Hu et al. [6] performed beam and pushout experimental tests to investigate longitudinal shear behavior of SCC beams. The authors accounted different parameters including: transverse reinforcement steel ratio, shear connection degree, longitudinal and transverse row numbers. The authors indicated failure modes of SCC beam were governed by transverse reinforcement steel ratios and degree of connections.

Numerical study on behavior of composite steel-concrete beam curved in plan loaded with monotonic load was evaluated by Jaafer and Saba [7]. The authors developed FE model by using a high-fidelity physics-based FEA program ABAQUS and the model was validated with experimental data in terms of its respective load-deflection curve, ultimate load, ultimate and yield deflection, and crack patterns. Jaafer and Saba [7] also conducted a parametric study to examine effects of beam span/radius ratio, different web stiffeners, partial interaction, concrete and steel material strengths. The authors showed span/radius ratio significantly influences curvature, web stiffeners affect propagation of shear stresses, and steel beam material strength immensely affects beam capacity. Similarly, Ismail et al. [8] numerically investigated effect of various parameters on castellated beams subjected to a vertical load. The authors concluded ultimate load of a castellated beam increased by using different vertical stiffeners around openings and decreasing slab slenderness. Moreover, Liu et al. [9] performed experimental and numerical studies to evaluate flexural strength of simply supported SCC beams under monotonic loading system. The authors also employed analytical formula from three building code of standards (namely: GB 50017, Eurocode 4, and BS 5950) to predict flexural response of SCC beam. The authors stated China’s code of standards gives better estimations as compared to the other two.

Previous numerical studies [10, 11] characterized on response of SCC beams when imposed with fatigue loads. El-Zohairy et al. [10] showed a systematic approach that can be used to enhance strength of composite system such as pre-compressing steel bottom flange to minimize tension portion of stress limit which in turn improves load carrying capacity of a SCC beam. Also, Deviyathi and Mohan [11] indicated composite beams are unlikely to fail in shear even under low shear span to effective depth ratio when subjected to increased number cycles of reverse loading conditions.

Previous studies also reported on effect and mitigation techniques of dynamic loads on civil engineering structures. Mohammed and Parvin [12, 13] studied nature of impact load on beam and bridge piers. The researchers investigated the response and performance of composite strengthened concrete structures when subjected to dynamic impact loadings. The authors' nonlinear FEA result revealed optimal ways of minimizing effects of impact loads including U shaped CFRP wrap techniques. Moreover, researchers [14, 15] studied nonlinear response of RC structural members under action of impact loading. The authors numerically investigated nonlinear behavior of RC structures subjected to low-velocity drop weight impact loading. The authors concluded LS-DYNA Winfrith concrete material model in very good accuracy predicted transient acceleration time histories and crack damage patterns. Various authors [16, 17, 18] also implemented a nonlinear FEA approach to study impact load response of RC slabs under varied drop-weight velocities, impact forces, energy capacities. The authors results confirm concrete damage models were well suited to capture dynamic impact loads response, displacement time history and concrete damage profiles.

Aforementioned survey of literatures on dynamic responses of steel concrete composite beam under synergetic effects of combined blast-impact loads indicate perceived gaps and meagre of researches on study of dynamic responses steel concrete composite structures when subjected to simultaneous loading of blast induced shock waves and various initial impactor velocities. The present study fills in perceived void in literature by presenting in depth study on response of SCC beam specimen subjected to combined blast-impact loading with various parameters including varied material strength and hysteretic properties [19], various cross section details of structural steel beam, impactor initial velocity, and reinforcement ratios.

Moreover, FEA parametric studies performed using a nonlinear program LS- DYNA accounting various material strengths, H-type structural steel beam flange and web thicknesses accompanied by numerous impacting initial velocities. Also, a free-air burst type blast loading, and drop weight impact loading with an initial velocity were deployed to simulate combined blast impact loading. Detailed FEA procedures and results are presented in following sections.

## 2 Description of validated experimental work

Recent experimental work reported by Hu et al. [6] was used for validation of developed finite element models. The experimental work was performed on steel-concrete composite (SCC) beam and focuses on the longitudinal shear behavior of SCC beams with longitudinal double-row studs. Next, details of the experiment including geometry, loading, boundary condition, material properties are presented.

### 2.1 Specimen details

**Figure 1:**
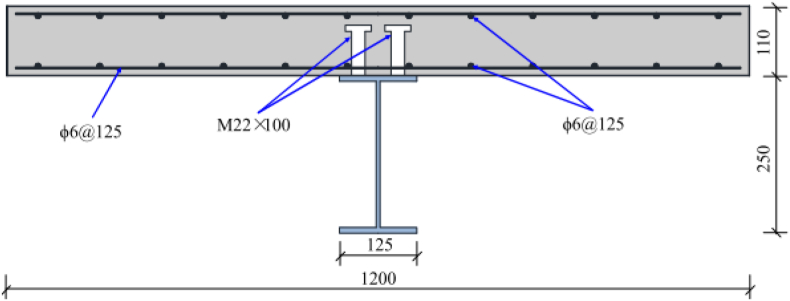
Cross-section of NCB-8 SCC beam specimen [6] (unit: mm).

**Figure 2:**
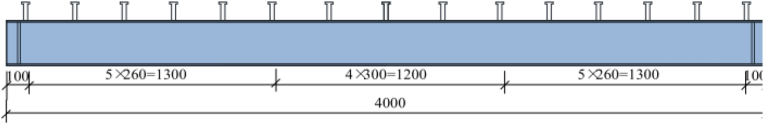
Side view of NCB-8 SCC beam specimen [6](unit: mm).

NCB-8 was one of the SCC beams that was tested by Hu et al. [6] to study the longitudinal shear behavior of SCC beams. The experimental findings of this beam were used to validate developed FEA models. The specimen was constructed from a reinforced concrete slab with a 1000 mm length and 600 mm width connected to a rolled steel beam (H-Section) with a section of beam depth of 250 mm, 125 mm flange width, web thickness of 6 mm, and 9 mm flange thickness. Headed studs were welded in a double row to top surface of a steel flange with diameter and length of 22 mm and 110 mm respectively. Detailed geometrical parameters of NCB-8 SCC beam are presented in Table 1 and Figures 1 and 2.

**Table 1:** Geometric parameters for NCB-8 specimen [6].

Parameter	Designation	Value
Span length	L	3800 mm
Steel beam type	H-Section	250x125 × 6 × 9 (mm)
RC slab width	B_c_	1200 mm
RC slab thickness	H_c_	110 mm
Stud diameter	D	22 mm
Stud length	H	100 mm
Transverse spacing of studs	S_t_	80 mm
Longitudinal spacing of studs	S_l_	260 mm

M22 studs spaced in two spacing schemes were employed and both longitudinal and transverse reinforcement steel bars with 6 mm diameter & 440 MPa yield strength are used as a reinforcement for the concrete slab. Specified compressive strength of concrete slab was 30 MPa and yield strength of studs and steel beam was 547 MPa and 342 MPa, respectively. Further details of specimen NCB-8 SCC beam are presented in Figures 1 and 2.

## 3 FEA modelling of SCC beam

LS-DYNA software program was used for Finite element analysis and simulation of impact and blast loading where as geometry and mesh generation was performed using ANSYS LSDYNA software program.

### 3.1 Section and material models

The FEA model which was used for validations (SCC_NCB_8) was coded and generated by using ANSYS LSDYNA software program. During this phase, 3D CPT 215 brick element model which has a 3-D eight-node coupled physical solid element capable of modeling coupled physical phenomena such as structural-fluid interaction and prevent volumetric mesh locking in nearly incompressible cases was employed. Furthermore, in order to overcome numerical instability and pathological mesh sensitivity to which strain-softening materials susceptible CPT215 element model was coupled with damage-plastic microplane models (see Tables 2 and 3). The slab was modeled as a thin shell using SHELL181 element. This element has compatible for analyzing thin to moderately-thick shell structures. The element has four nodes with six degrees of freedom at each node which is compatible with aforementioned CPT 215 element.

**Table 2:** Elastic microplane element properties.

Parameter	Symbol	Description	Value
Elastic	E	Modulus of elasticity	200 MPa
	ѵ	Poisson ratio	0.18

**Table 3:** Plastic microplane element properties.

Parameter	Symbol	Description	Value
	f_cu_	Uniaxial compressive strength	26.775 MPa
	f_bc_	Biaxial compressive strength	30.79 MPa
	f_bt_	Uniaxial tensile strength	2.699 MPa
Plastic	R	Ratio between major & minor axes of the cap	1
	D	Hardening material constant	4000
	R_T_	Tension cap hardening cap	1

REINF264 element was used to model reinforcement bars. The element is suitable for simulating reinforcing fibers with arbitrary orientations. Since reinforcement bar in reinforced concrete and composite structures is mainly employed to resists tensile or compressive state of stress, each fiber in this study, is modeled separately as a spar that has only uniaxial stiffness. Tables 4, 5, and 6 depicts material properties for reinforcement bars, H-Section structural steel and M22 stud structural elements, respectively.

**Table 4:** Reinforcement bar material properties.

Symbol	Description	Value
E	Modulus of elasticity	223000 MPa
ѵ	Poisson ratio	0.3
f_yk_	Yield strength	440 MPa

**Table 5:** H-section structural steel material properties.

Symbol	Description	Value
E	Modulus of elasticity	223000 MPa
ѵ	Poisson ratio	0.3
f_yk_	Yield strength	342 MPa

**Table 6:** M22 stud steel properties.

Symbol	Description	Value
E	Modulus of elasticity	223000 MPa
ѵ	Poisson ratio	0.3
f_yk_	Yield strength	547 MPa

First phase FEA modelling technique analysis was employed by implicit static analysis technique using ANSYS LS-DYNA software program. This is followed by explicit FEA using LS-DYNA software program by adopting only quantities evaluated at time steps preceding time t + Δt Vaiana et al. [20].

For solid sections of validated FEA model (SCC_NCB_8), a constant stress solid element formulation with 8-noded hexahedron element having twenty-four degrees of freedom was employed. The formulation of this element was done by attaching an iso-parametric (natural) coordinate system to the element. Moreover, for solid section material, a continuous surface cap material (CSCM) model which is designed for blast and impact loads was deployed. CSCM was automatically generates the required parameters with entry of unconfined compressive strength of a concrete (FPC = 31.5 MPa) and concrete mass density (RO = 2.7e − 09 tonnes/mm3). Also, erosion feature this element is activated by inputting erosion factor (ERODE = 1.05).

Since reinforcement steel bars resist either tensile and/or compressive stresses, a truss (bar) section model was used for both longitudinal and transverse reinforcement bars embedded in the concrete slab above H-type steel beam. Elastic plastic with kinematic hardening material model with activated rate effect employed to characterize reinforcement steel bar [21]. For reinforcement steel bars, H-type steel beams, and studs, a mass density (RO = 7.85e − 09 tonnes/mm^3^), Young’s modulus (E = 2.23e + 05 MPa), Poisson’s ratio (PR = 0.3), Hardening parameter (BETA = 1.0) were used whereas, yield stresses of (SIGY = 440 MPa, 342 MPa, 547 MPa) were used for reinforcement steel bars, H-type structural steel beam, and studs respectively.

### 3.2 Boundary conditions and use of symmetry

**Figure 3:**
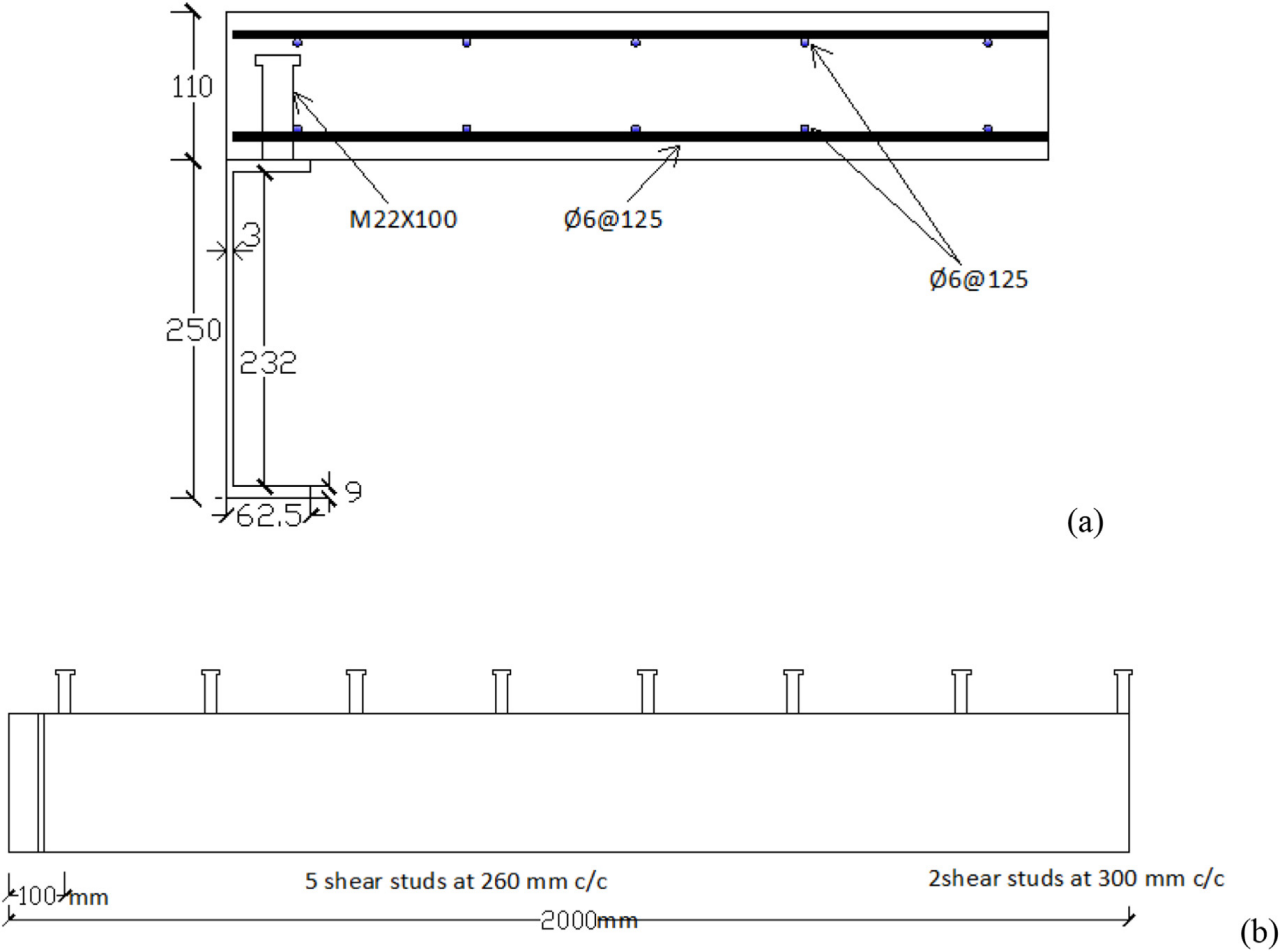
Quarter size details of SCC beam (a) cross-section; and (b) longitudinal views.

A nodal point constraint (∗SPC) was used in a local system to apply boundary condition in developed FE model. In addition to this, appropriate use of symmetry was employed to reduce computational demand. At plan of symmetry, displacement boundary condition perpendicular to the plane of symmetry was set to zero. Thus, one can use a finer subdivision of elements with less computational cost, time of analysis, and modeling effort [22]. Similarly, in the present study taking advantage of symmetry, FE model was developed considering only quarter size model of a SCC beam (see Figure 3). Boundary conditions were prescribed to prevent rigid body motion of a beam that may be induced during combined blast-impact loading.

### 3.3 Loading conditions

Self-weight of the SCC beam was taken into account by using ∗LOAD_BODY keyword commands. A load curve scale factor for gravitational acceleration (SF = −9810 mm/s^2^) was applied where the negative sign indicating downward direction of forces.

An enhanced blast load (∗LBE) keyword command was used to simulate blast loading scenarios. The only mandatory input parameters in ∗LBE keyword were equivalent TNT charge weight (M), location of charge mass center (XBO, YBO, ZBO), unique blast id (BID), type of blast source (BLAST), and typical segment (SEGMENT_SET). A spherical free-air burst on with no amplification of initial shock wave due to interaction with ground surface was allowed to hit top surface of the slab.

The impact load was simulated by giving an initial velocity (∗INITIAL) to a box-like drop weight. BOXID key word command was employed to initialize all nodes in the box initial velocity in Y-direction (VY = -3402 mm/s). The negative sigh represents downward direction of drop weight.

### 3.4 Displacement and effective stress-strain monitoring points

**Figure 4:**
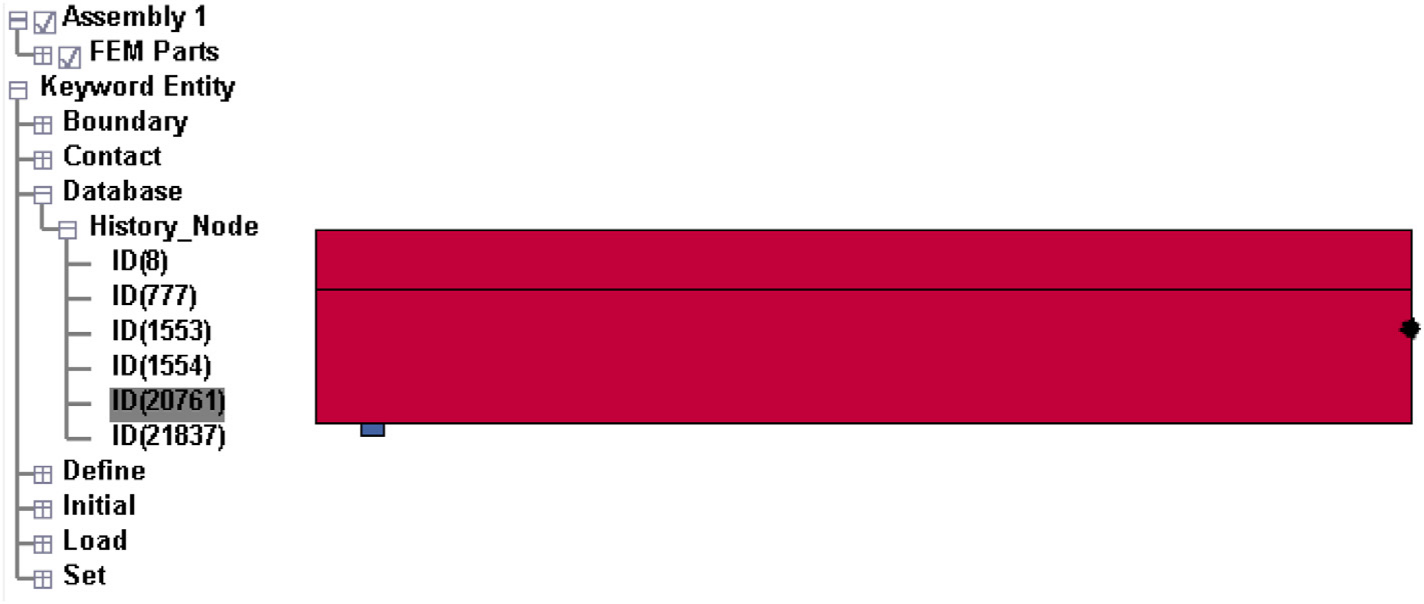
Displacement-time history monitoring point.

**Figure 5:**
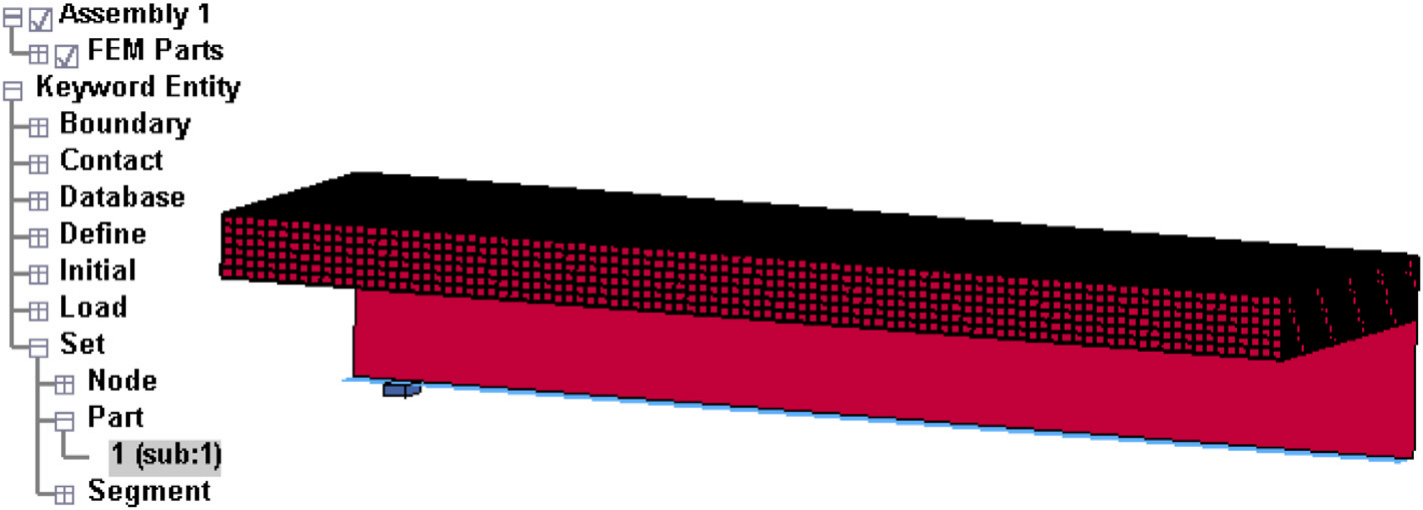
Concrete deck part used for gauging effective plastic strain.

**Figure 6:**
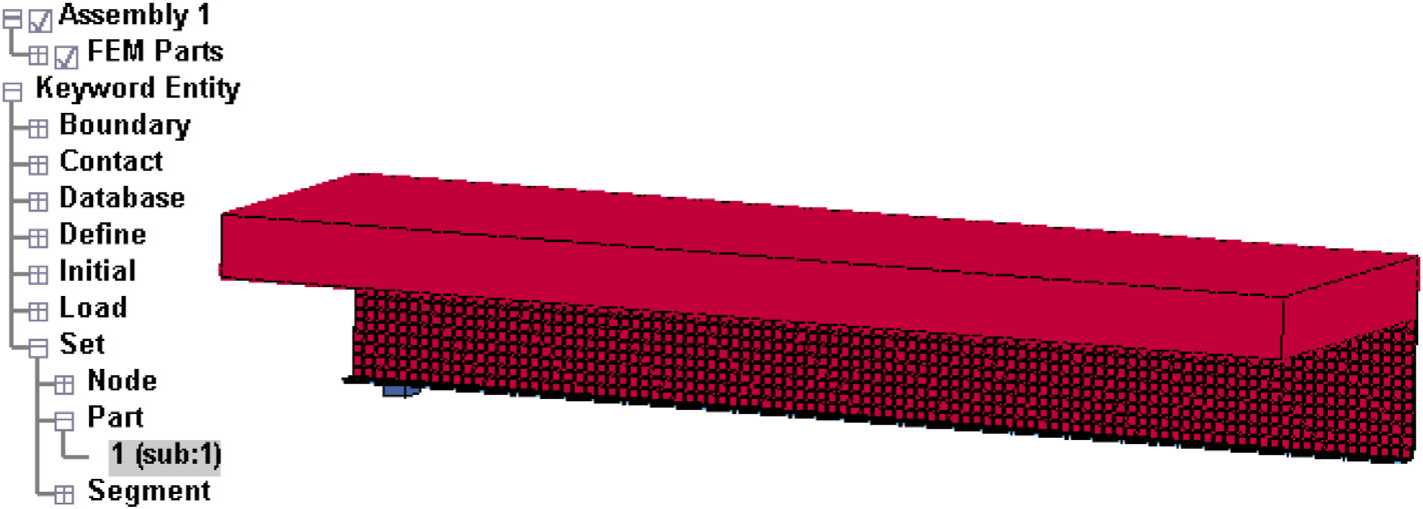
Steel beam part used for gauging effective stress.

Displacement time history output was extracted by issuing LS-DYNA ASCII_NODOUT command. Beam nodes at impact location were selected to monitor Displacement time history output (Figure 4). Similarly, Von Mises stress and effective plastic strain were measured from concrete deck element and H-type structural steel element, respectively (Figures 5 and 6).

### 3.5 Bonding

Since in SCC beam, bonding exists between the reinforcement bar and concrete and also a bond between steel top flange and concrete slab, an effective bonding between those parts is utilized by using LSDYNA’s CONSTRAINED_LAGRANGE_IN_SOLID command.

**Figure 7:**
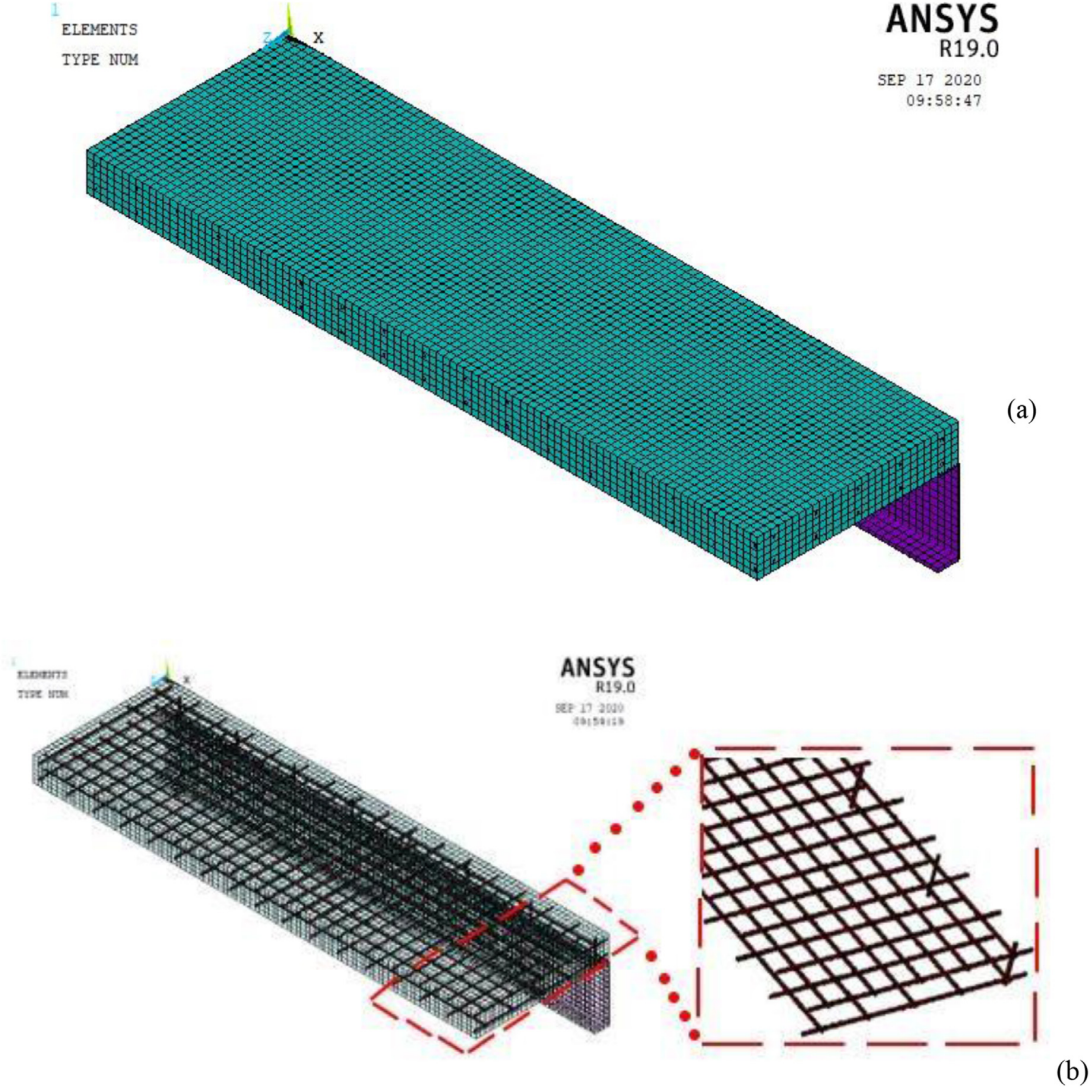
ANSYS Mechanical APDL quarter size reduced SCC beam FEA model: (a) 3D model with mesh; and (b) reinforcement details.

**Figure 8:**
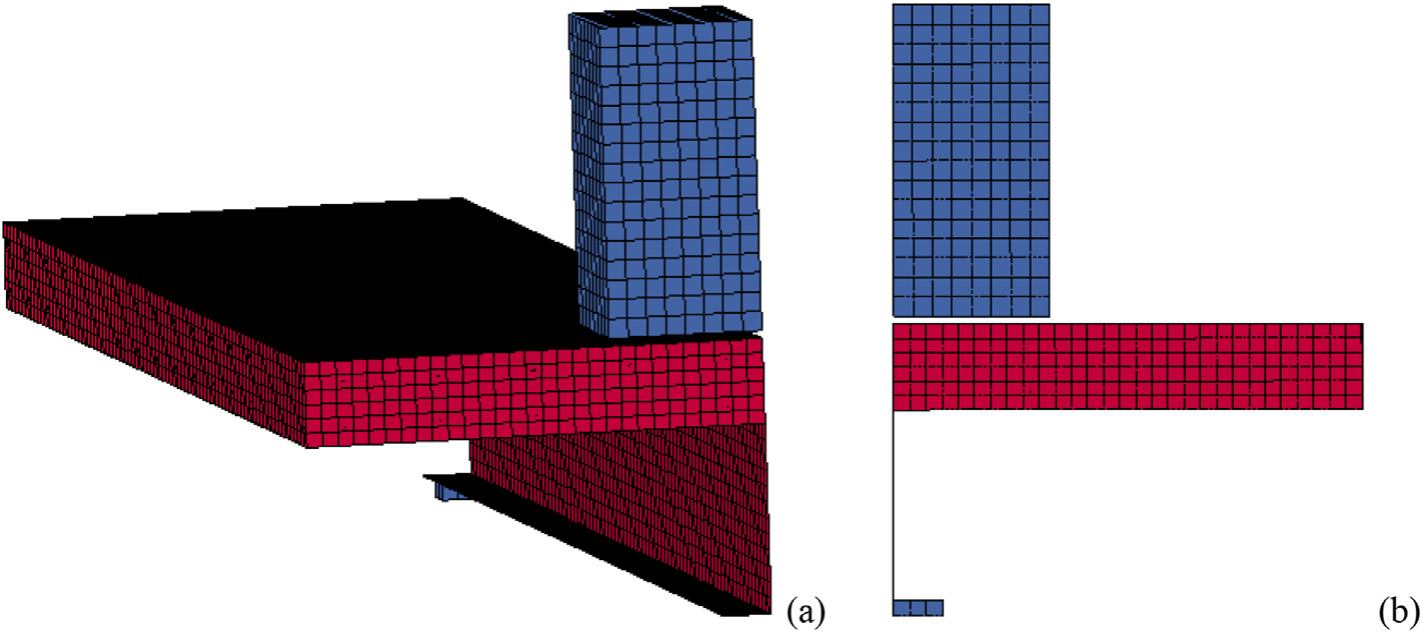
LS-DYNA quarter size reduced SCC beam FEA model with location of impactor: (a) 3D view; and (b) side view.

Figures 7 and 8 displays concrete, H-Section structural steel, reinforcement bars, M22 stud steels, and impactor details of developed and validated FEA model (SCC_NCB_8).

### 3.6 Finite element analysis results

#### 3.6.1 FE validation analysis results

**Figure 9:**
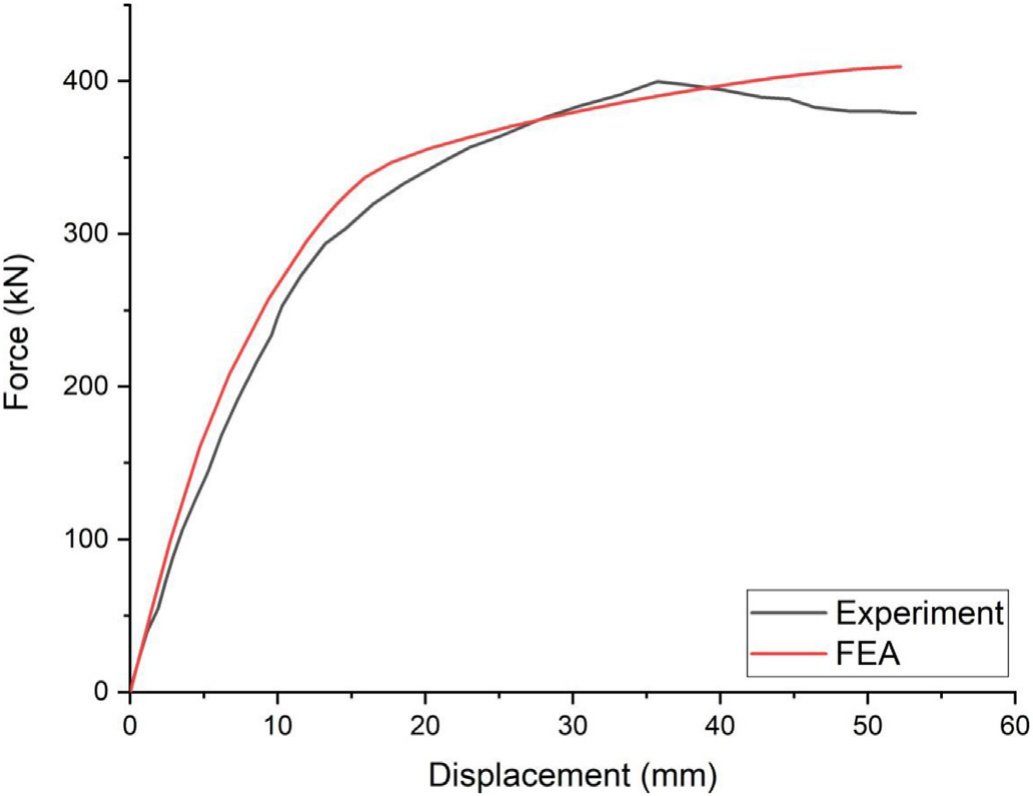
Comparison of load-deflection curve for experiment and FEA.

Figure 9 presents load versus displacement plots of FE validation model (SCC_NCB_8) and experimental results of test specimen (NCB_8) [6]. Overall, FE validation result plot general behavior matches well with experimental test result.

Percentage of difference between peak FE and experimental result is 2.4%. Moreover, as compared to experimental result, FE load deflection plot shows slightly stiffer this might be due to perfect bond assumption between concrete and rebar steel; and connection between concrete, studs and steel beam.

**Figure 10:**
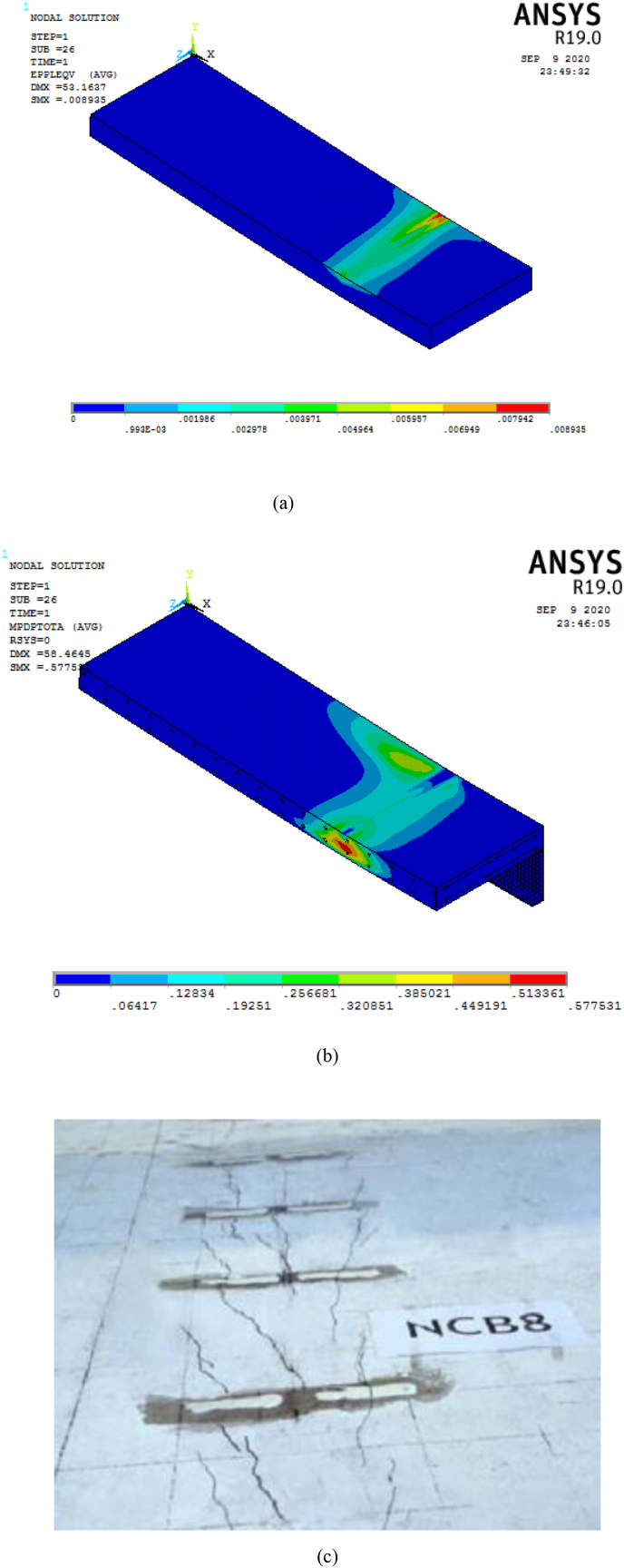
FE validation longitudinal shear failure and crack pattern results: (a) Major crack pattern; (b) Minor crack pattern; and (c) experiment Hu et al. [6].

Figure 10 displays longitudinal shear failure of SCC beam which was typically caused by stud shearing. The main reason for this type of failure was that NCB-8 SCC beam had partial shear connection which permits transverse cracks to appear on lower surface of the RC slab at exact loading point, and the cracks extended continuously progressed into top of RC slab. Moreover, crack propagation of FE validation results for specimen (SCC_NCB_8) showed good agreement with experimental result (Figure 10). A good matching observation between finite element validation analysis results and experimental results of load deflection plot (Figure 9) and crack pattern (Figure 10) implies accuracy and reliability of developed finite element model.

### 3.7 FE parametric study

**Figure 11:**
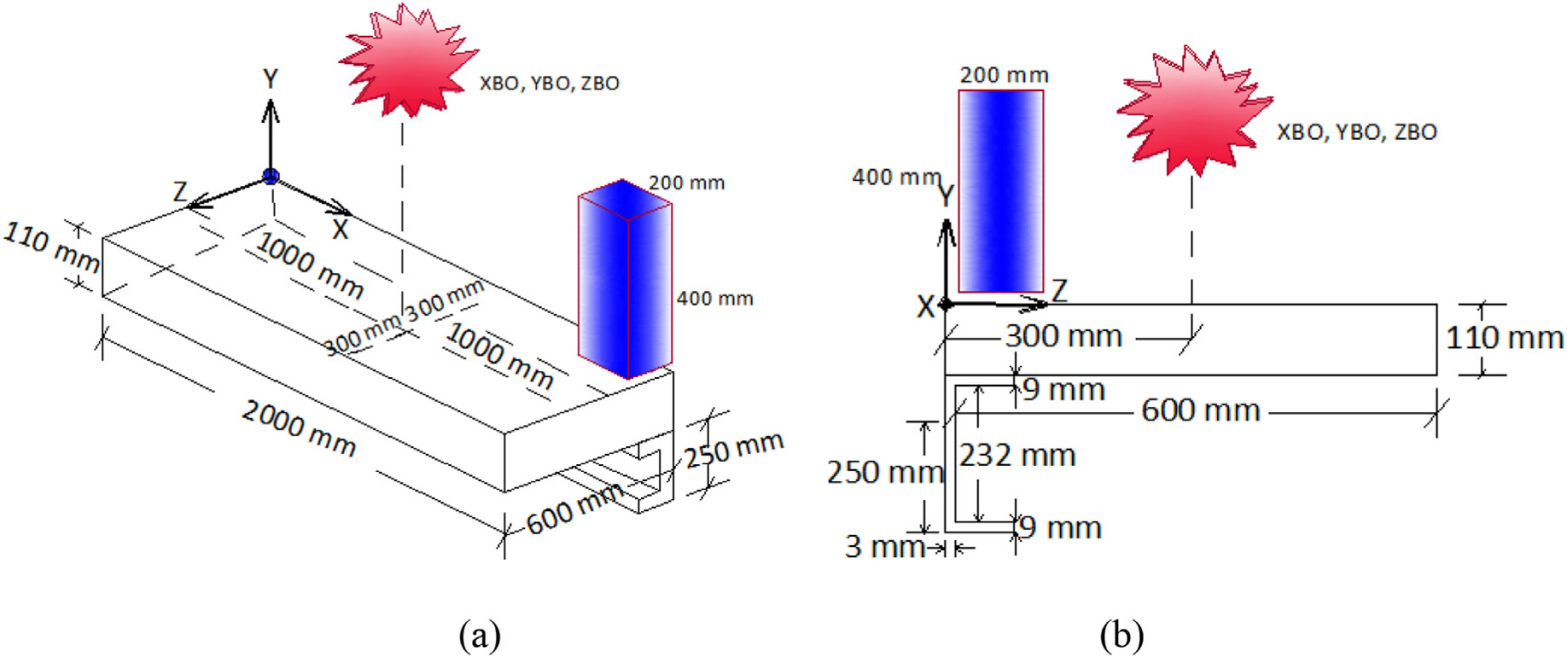
Sketches for blast origin coordinate in X, Y and Z axis: (a) 3D view; and (b) side view.

**Figure 12:**
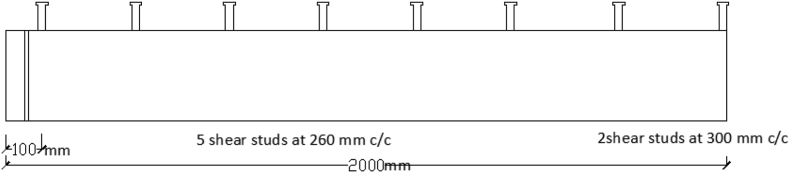
Arrangement of stud numbers and spacings used for parametric study.

Based on validated FEA model, a parametric study on dynamic responses of SCC beam under combined blast-impact loading was further carried out to investigate influence of different parameters on material strengths and section properties with various impactor velocities. Considered blast loading was a spherical free air bursts with initial blast induced shockwave being not amplified by ground surface. Explosive positions were set by blast origins: XBO, YBO, and ZBO (see Figure 11). For all blast scenarios, stud numbers and spacings remain constant throughout the analysis (see Figure 12).

In first FE parametric study, various values of characteristics compressive strength of concrete (confirming to Tabl 3.1 of Eurocode-2 [23]) were used to study effect of different concrete strengths on SCC beam under combined blast and impact loads. Also, specified yield stress for reinforcement bars, studs, and steel beam were 440 MPa, 547 MPa, and 342 MPa respectively similar to validation FE.

In second FE parametric study, compressive strength of concrete 30 MPa remained constant and then various values of specified yield stress of structural steel and studs (confirming to Tabl 3.1 of Eurocode-3 [24]) were employed to study effect of different specified yield stresses of steel beam and studs. Also, effect of different flange and web thicknesses on the dynamic response of SCC beam were also studied accordingly.

In third FE parametric study, compressive strength of concrete 30 MPa remained constant; yield stress of H-Section structural steel beam and studs were set 342 and 547 MPa respectively and various values of specified yield stress of reinforcement bars (confirming to Section 3.2 of Eurocode-2 [23]) were used to study effect of different reinforcement strengths of reinforcement steel rebars. Parametric study details such as effect of compressive strength of concrete, yield stress of H-Section steel beam, and studs used in present study are shown in Tables 7, 8, 9, 10, 11, and 12.

**Table 7:** Blast-impact scenario-A.

Effect of Different Specified Compressive Strengths of Concrete								
Blast scenario	Compressive strength of Concrete	Enhanced Blast Load Property						
	^*f*^*ck* (MPa)	BT	CHM (kg)	NEGPHS	XBO (mm)	YBO (mm)	ZBO (mm)	Z $\left(\frac{m}{kg^{\frac{1}{3}}}\right)$
1A	20	Free-air	12	ON	1000	3500	300	1.53
2A	30	Free-air	12	ON	1000	3500	300	1.53
3A	40	Free-air	12	ON	1000	3500	300	1.53

**Table 8:** Blast-impact scenario-B.

Effect of Different Specified Yield Stresses of H-Section Structural Steel								
Blast scenario	Yield Stress of Structural Steel	Enhanced Blast Load Property						
	*f*_*y*_ (MPa)	BT	CHM (kg)	NEGPHS	XBO (mm)	YBO (mm)	ZBO (mm)	Z $\left(\frac{m}{kg^{\frac{1}{3}}}\right)$
1B	235	Free-air	20	ON	1000	3000	300	1.11
2B	275	Free-air	20	ON	1000	3000	300	1.11
3B	355	Free-air	20	ON	1000	3000	300	1.11

**Table 9:** Blast-impact scenario-C.

Effect of Different Specified Yield Stresses of Studs								
Blast scenario	Yield Stress of Stud	Enhanced Blast Load Property						
	*f*_*y*_ (MPa)	BT	CHM (kg)	NEGPHS	XBO (mm)	YBO (mm)	ZBO (mm)	Z $\left(\frac{m}{kg^{\frac{1}{3}}}\right)$
1C	460	Free-air	25	ON	1000	6000	300	2.05
2C	540	Free-air	25	ON	1000	6000	300	2.05
3C	600	Free-air	25	ON	1000	6000	300	2.05

**Table 10:** Blast-impact scenario-D.

Effect of Different Specified Yield Stresses of Reinforcement Steel Bars								
Blast scenario	Yield Stress of Rebar	Enhanced Blast Load Property						
	*f*_*y*_ (MPa)	BT	CHM (kg)	NEGPHS	XBO (mm)	YBO (mm)	ZBO (mm)	Z $\left(\frac{m}{kg^{\frac{1}{3}}}\right)$
1D	400	Free-air	12.5	ON	1000	4000	300	1.72
2D	440	Free-air	12.5	ON	1000	4000	300	1.72
3D	500	Free-air	12.5	ON	1000	4000	300	1.72

**Table 11:** Blast-impact scenario-E.

Effect of Different Flange Thickness of H-type Structural Steel Beam								
Blast scenario	Flange Thickness	Enhanced Blast Load Property						
	t_f_ (mm)	BT	CHM (kg)	NEGPHS	XBO (mm)	YBO (mm)	ZBO (mm)	Z $\left(\frac{m}{kg^{\frac{1}{3}}}\right)$
1E	3	Free-air	10	ON	1000	4000	300	1.86
2E	5	Free-air	10	ON	1000	4000	300	1.86
3E	7	Free-air	10	ON	1000	4000	300	1.86

**Table 12:** Blast-impact scenario-F.

Effect of Different Web Thickness of H-type structural Steel Beam								
Web Thickness		Enhanced Blast Load Property						
Blast scenario	t_w_ (mm)	BT	CHM (kg)	NEGPHS	XBO (mm)	YBO (mm)	ZBO (mm)	Z $\left(\frac{m}{kg^{\frac{1}{3}}}\right)$
1F	9	Free-air	15	ON	1000	5000	300	2.03
2F	11	Free-air	15	ON	1000	5000	300	2.03
3F	13	Free-air	15	ON	1000	5000	300	2.03

### 3.8 Effect of varying compressive strength of concrete

Finite element analysis was performed to study effect of concrete grade on response of a SCC beam under combined blast-impact loading. Three strengths of concrete namely 20 MPa, 30 MPa, and 40 MPa were used in the FE study. Enhanced blast load with 12 kg charge mass and scaled distance Z = 1.53 $\left(\frac{m}{kg^{\frac{1}{3}}}\right)$ was applied on SCC beam.

**Figure 13:**
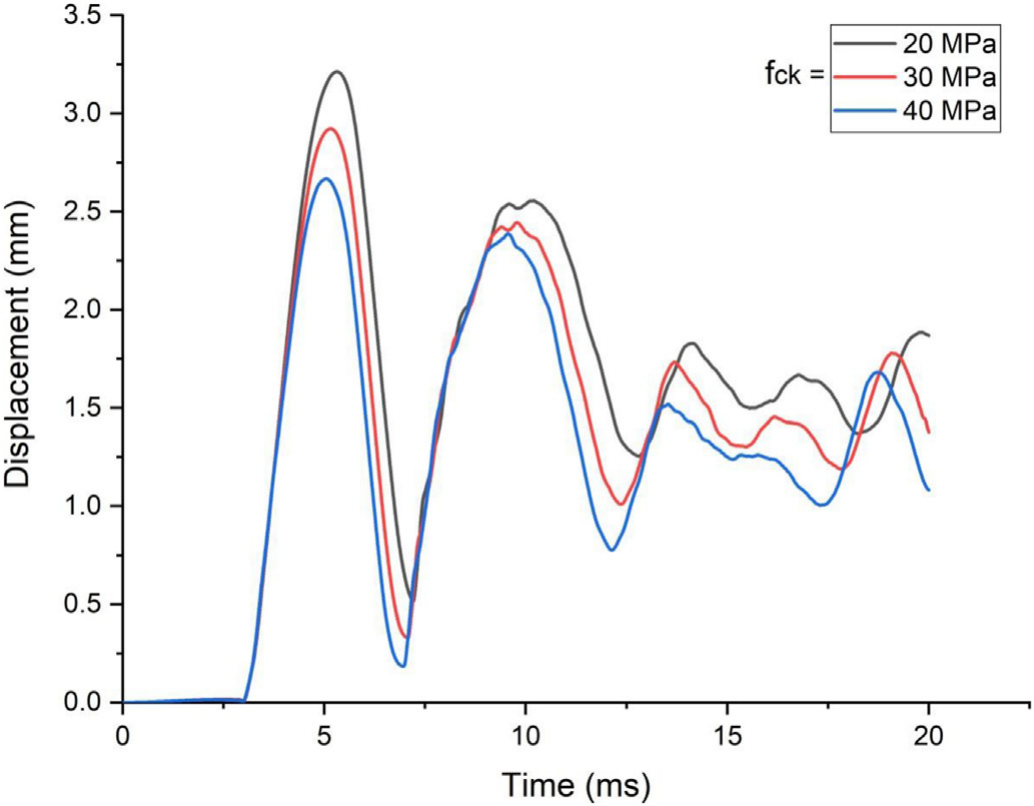
Displacement-time history plots for SCC beam with different specified strengths of concrete struck by 1.5 m/s impactor initial velocity.

**Figure 14:**
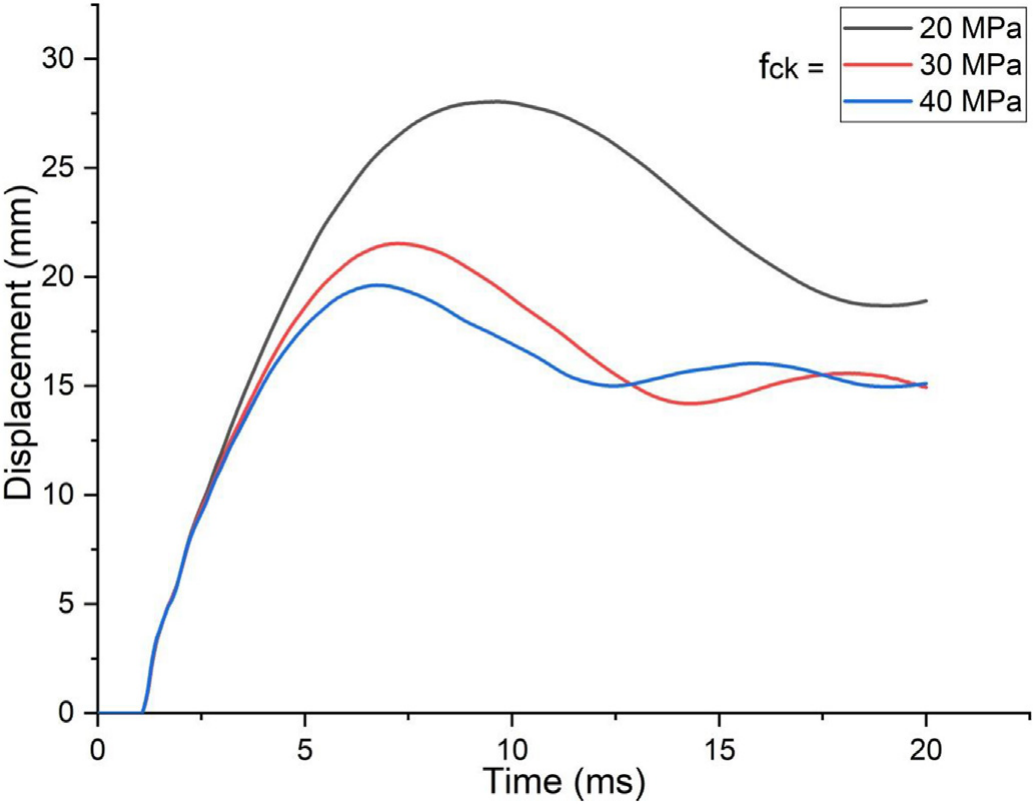
Displacement-time history curves for SCC beam with different specified strengths of concrete struck by 10 m/s impactor initial velocity.

The effect of grade of concrete comes in to picture as velocity of impactor increases from low to high impacting velocity (Figures 13 and 14). For 1.5 m/s low impact speed, as compared to 20 MPa SSC beam, 30 MPa and 40 MPa SCC beams exhibited 9% and 17% drop in maximum displacement where as for 10 m/s high impact speed, reduction in displacement values were 23 % and 30 % respectively.

**Figure 15:**
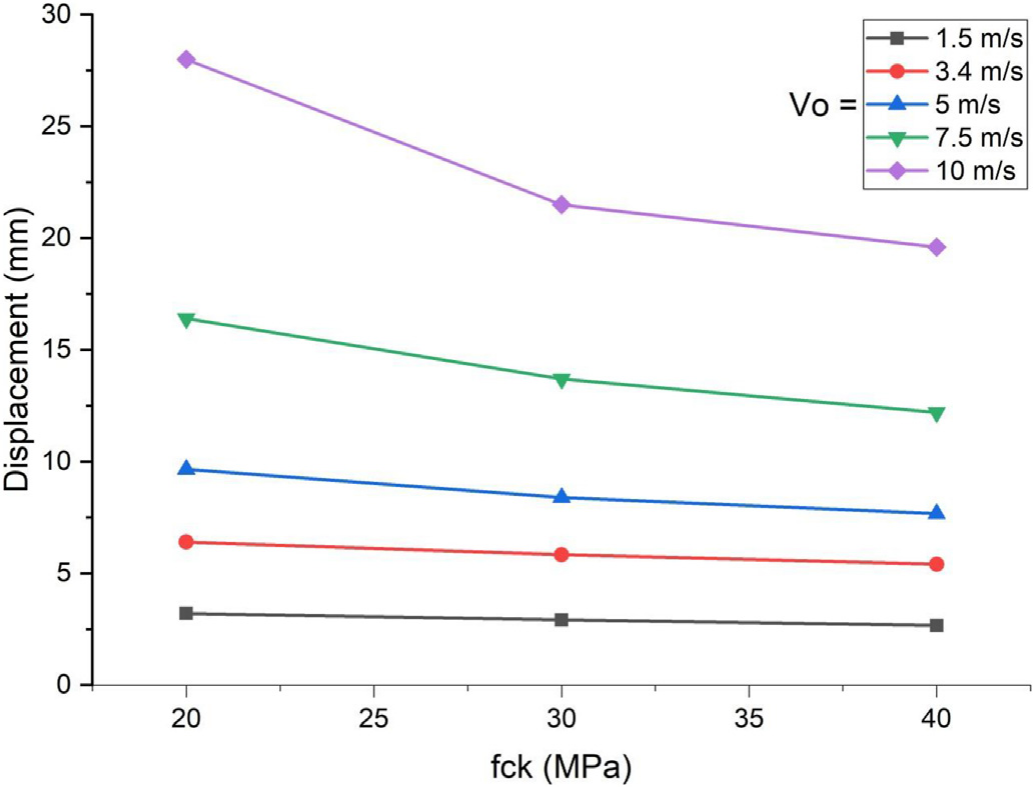
Comparison of displacement versus specified compressive strength of concrete (f_ck_) for SCC beam under various ranges of impactor initial velocities (V_o_).

Figure 15 shows similar trend where effect of concrete grade was insignificant for blast load combined with low impacting speed however as impacting speed threshold raised to 7.5 m/s and above, effect of concrete grade materialized significantly.

**Figure 16:**
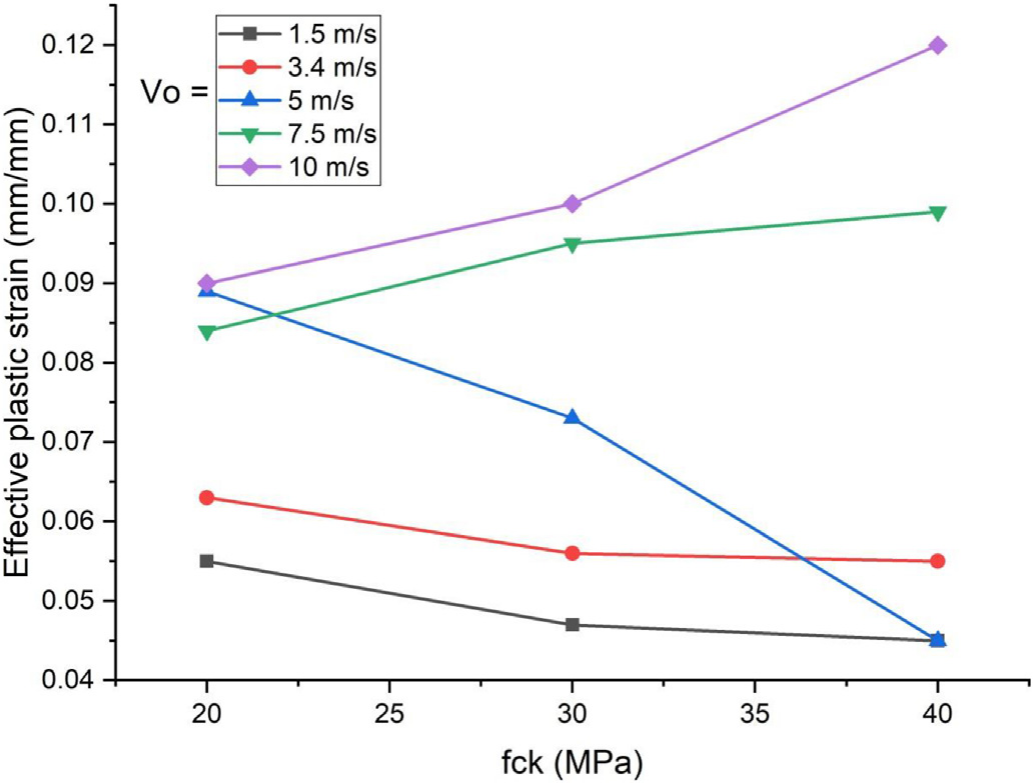
Comparison of effective plastic strain versus specified compressive strength of concrete (f_ck_) for SCC beam under various ranges of impactor initial velocities (V_o_)

As shown in Figure 16, use of blast-impact scenario with and below 5 m/s impactor initial velocity exhibited a reduced effective plastic strain values on concrete deck. Conversely in cases of blast loading combined impacting load with initial velocity of 7.5 m/s and above showed an increase in effective plastic strain values. This may be due to strain rate dynamic effect accompanied by an increase of material strength.

### 3.9 Effect of varying yield stress of H-type structural steel beam

In this parametric study, effect of different yield stresses of H-type structural steel beam was investigated by keeping other SCC beam characteristics such as span, reinforcement steel ratio, number of studs, spacing of studs, and other material strength constant. Enhanced blast load with 20 kg charge mass and scaled distance Z = 1.11 $\left(\frac{m}{kg^{\frac{1}{3}}}\right)$ was applied on SCC beam. Three different yield stresses of H-type structural steel beam namely 235 MPa, 275 MPa, and 355 MPa were considered in the current parametric study.

**Figure 17:**
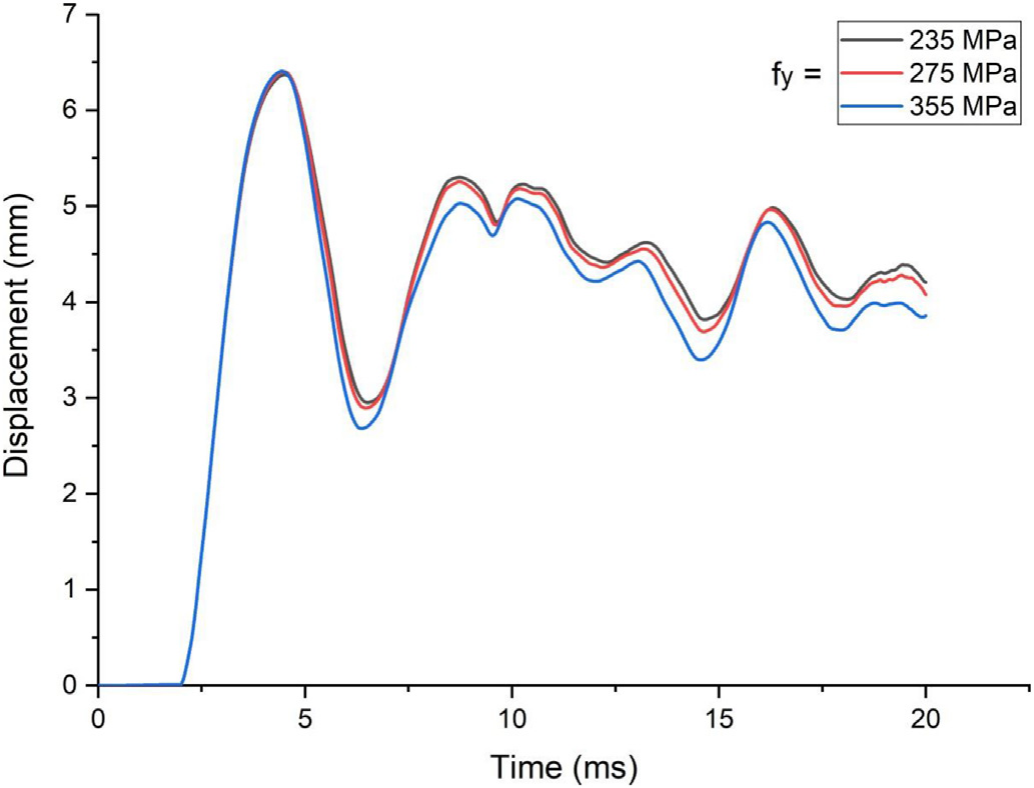
Displacement-time history plot of SCC beam with different yield stresses of H-type structural steel beam struck by 1.5 m/s impactor initial velocity.

Figure 17 presents displacement time history response of a SCC beam under blast loading combined with 1.5 m/s low velocity impact loading. Varying yield stresses of H-type structural steel beams from 235 MPa to 355 MPa under blast loading combined with 1.5 m/s low velocity impact loading showed insignificant effect in peak deflection values of SCC beam. Identical post peak deflection profiles were observed in SCC beam while varying yield stresses of H-type structural steel beams. This implies absence of any performance gain while varying yield stresses of H-type structural steel beams in cases of blast loading combined with low velocity impact loading.

**Figure 18:**
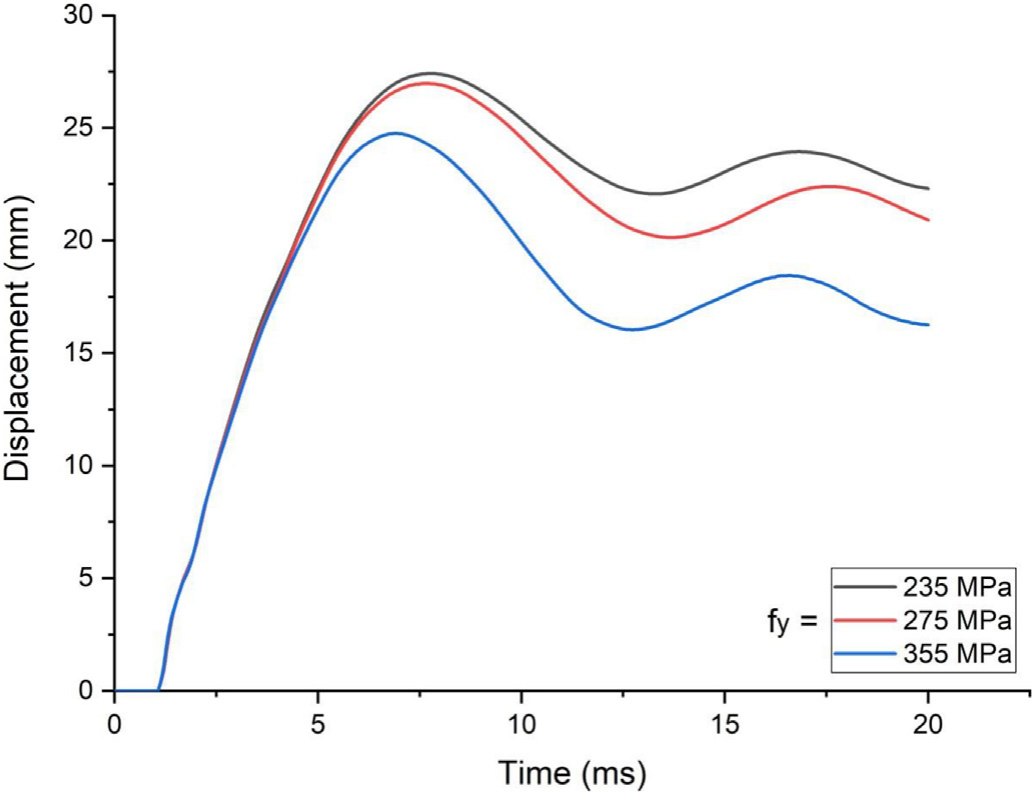
Displacement-time history plot of SCC beam with different yield stresses of H- type structural steel beam struck by 10 m/s impactor initial velocity.

**Figure 19:**
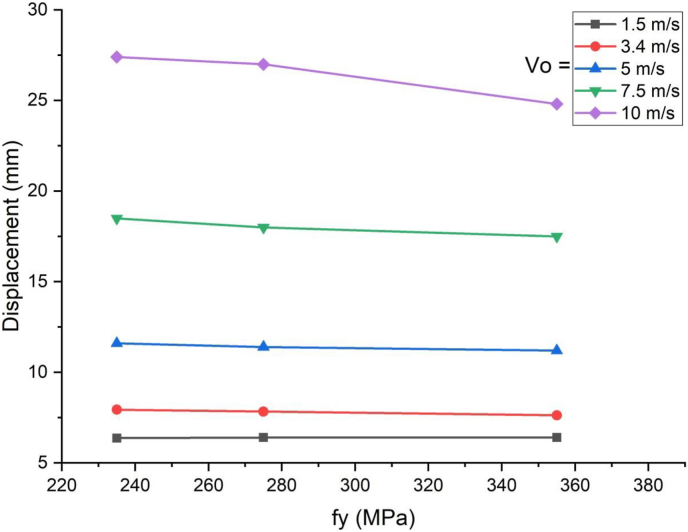
Comparison of displacement versus yield stresses of H-type structural steel beam (f_y_) for SCC beam under various ranges of impactor initial velocities (V_o_).

**Figure 20:**
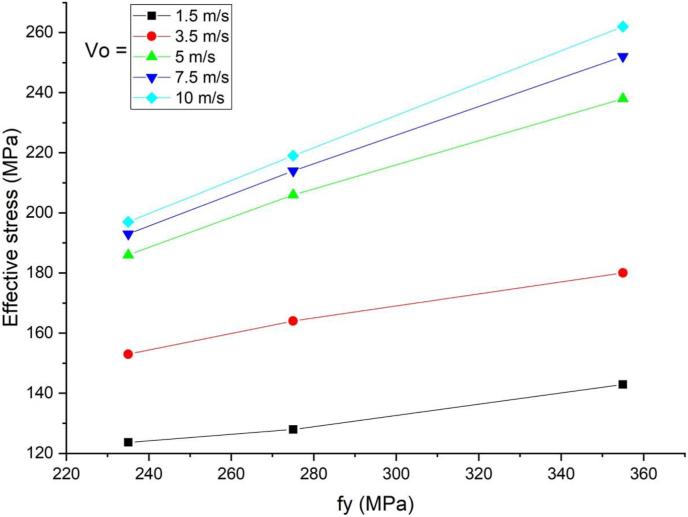
Comparison of effective plastic strain versus yield stress of H-type structural steel beam (f_y_) for SCC beam under various ranges of impactor initial velocities (V_o_).

In cases of blast load combined with high velocity impacting, load deflection plots in linear elastic ranges were identical for all H-type structural steel beams yield stresses values however stark differences in load deflection profiles emerge in nonlinear and post peak regions of load deflection plots (Figure 18). Similarly, substantial reduction in peak displacement values were as H-type structural steel beam yield stresses value was raised from 235 MPa to 355 MPa in blast loading combined with high velocity impacting scenarios (Figure 19). On the other hand, use of a blast-impact scenario with and below 3.4 m/s impactor initial velocity exhibited reduced effective plastic strain values and conversely a case of combined loading above 5 m/s showed an increase in the effective plastic strain values (Figure 20).

### 3.10 Effect of varying yield stress of studs

**Figure 21:**
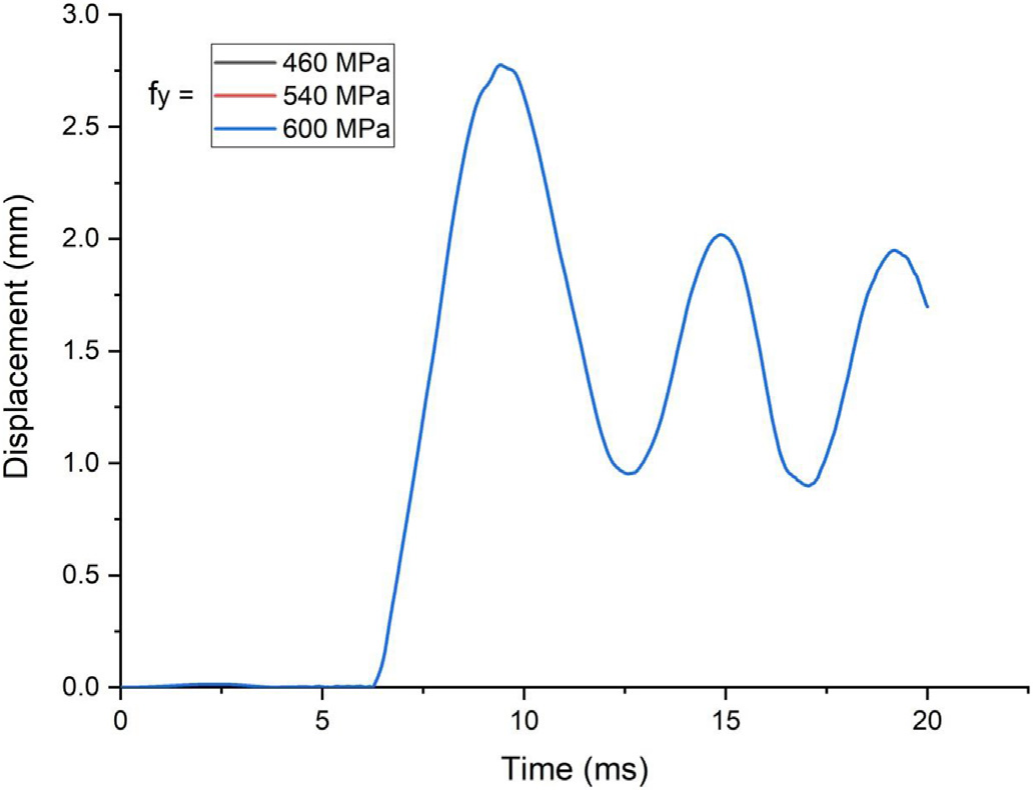
Displacement-time history plot for SCC beam with various yield stresses of studs struck by 1.5 m/s impactor initial velocity.

**Figure 22:**
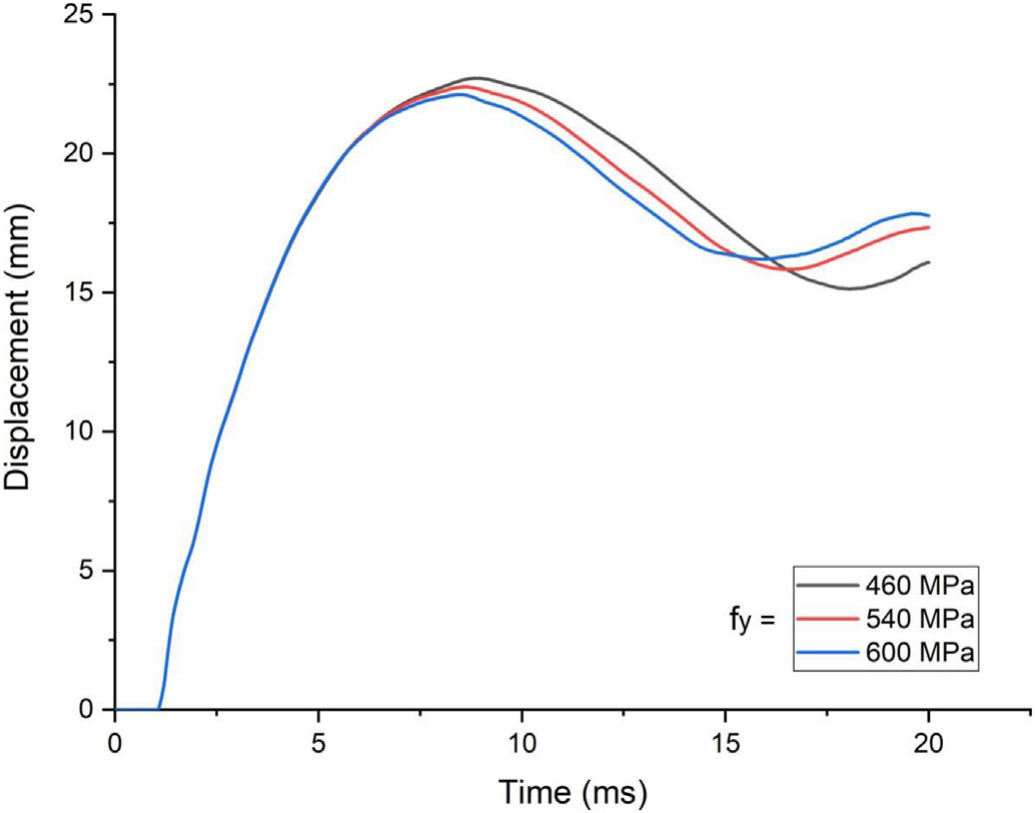
Displacement-time history plots for SCC beam with various yield stresses of studs struck by 10 m/s impactor initial velocity.

**Figure 23:**
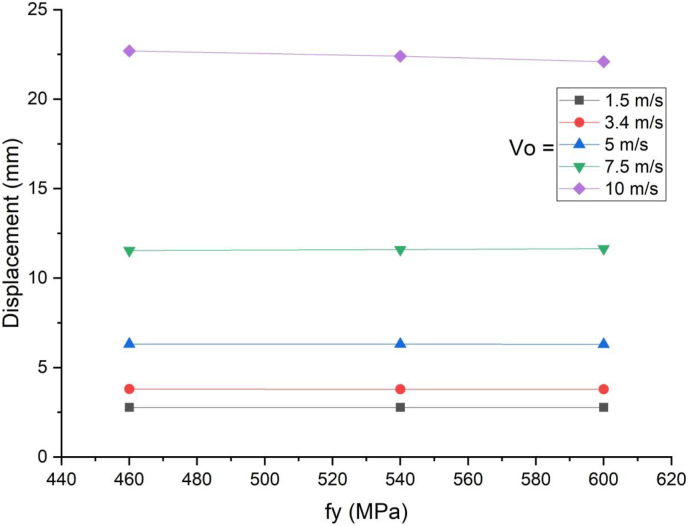
Comparison of displacement versus yield stresses of studs (f_y_) for SCC beam under various ranges of impactor initial velocities (V_o_).

**Figure 24:**
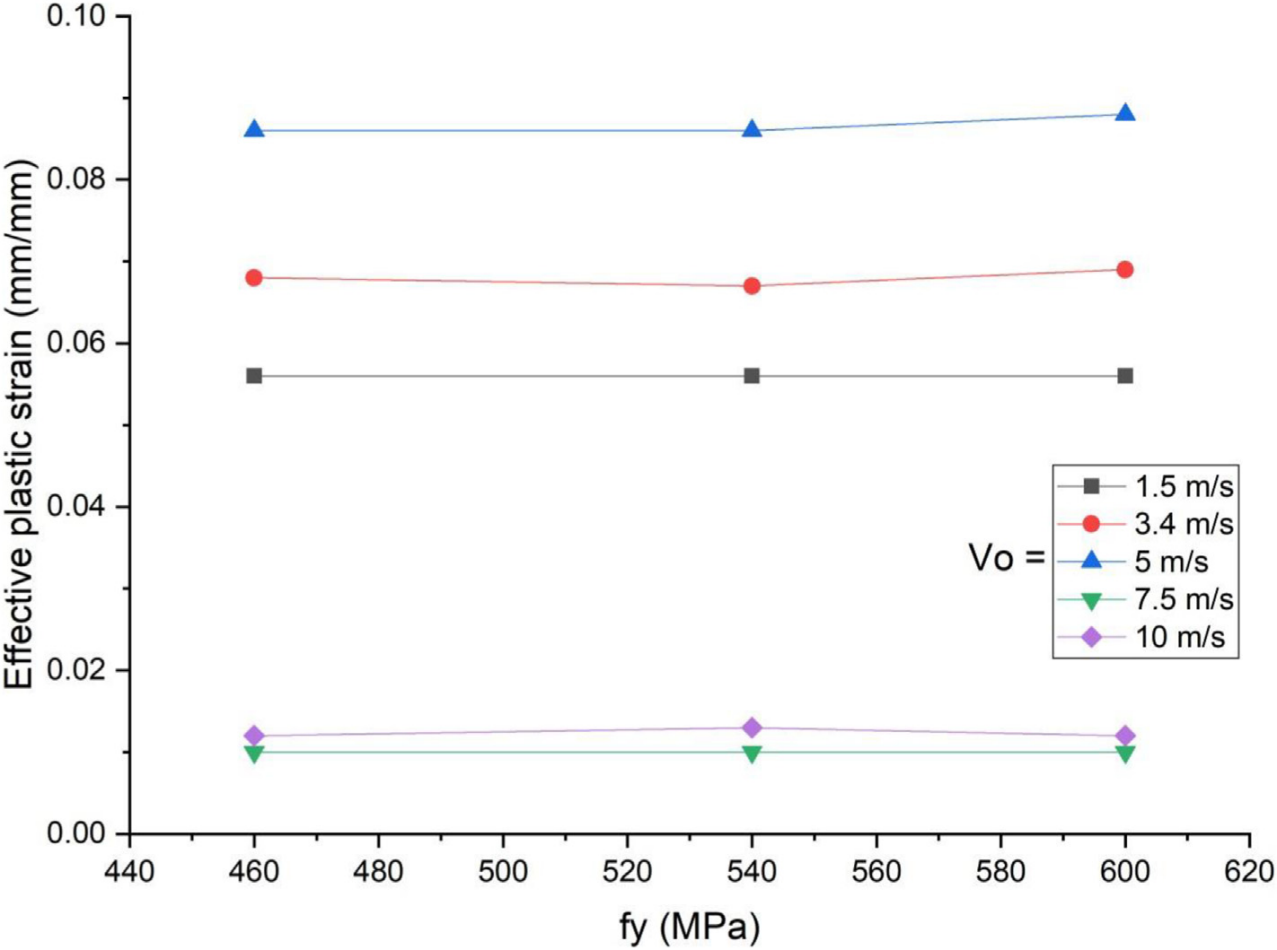
Comparison of effective plastic strain versus yield stress of studs (f_y_) for SCC beam under various ranges of impactor initial velocities (V_o_).

In this section, parametric study on use of studs in SCC beam with varying yield strength (460 MPa, 540 MPa, and 600 MPa) was investigated. An enhanced blast load with 25 kg charge mass and scaled distance Z = 2.05 $\left(\frac{m}{kg^{\frac{1}{3}}}\right)$ was applied on SCC beam. As shown in Figure 21, there was insignificant performance gains for varying stud yield strength of SCC beam subjected to blast load combined with low velocity impact loading. In linear elastic range of load deflection plot (Figure 22), similar trend was observed even for blast load combined with high velocity impact loading however this is changed in nonlinear and post peak region of load deflection where slight performance gains were observed. The same is true for effective plastic strain and peak displacement values, no visible net gains were observed for SCC beam under blast load combined with low and high impact velocity (Figures 23 and 24).

### 3.11 Effect of varying yield stress of reinforcements steel bars

**Figure 25:**
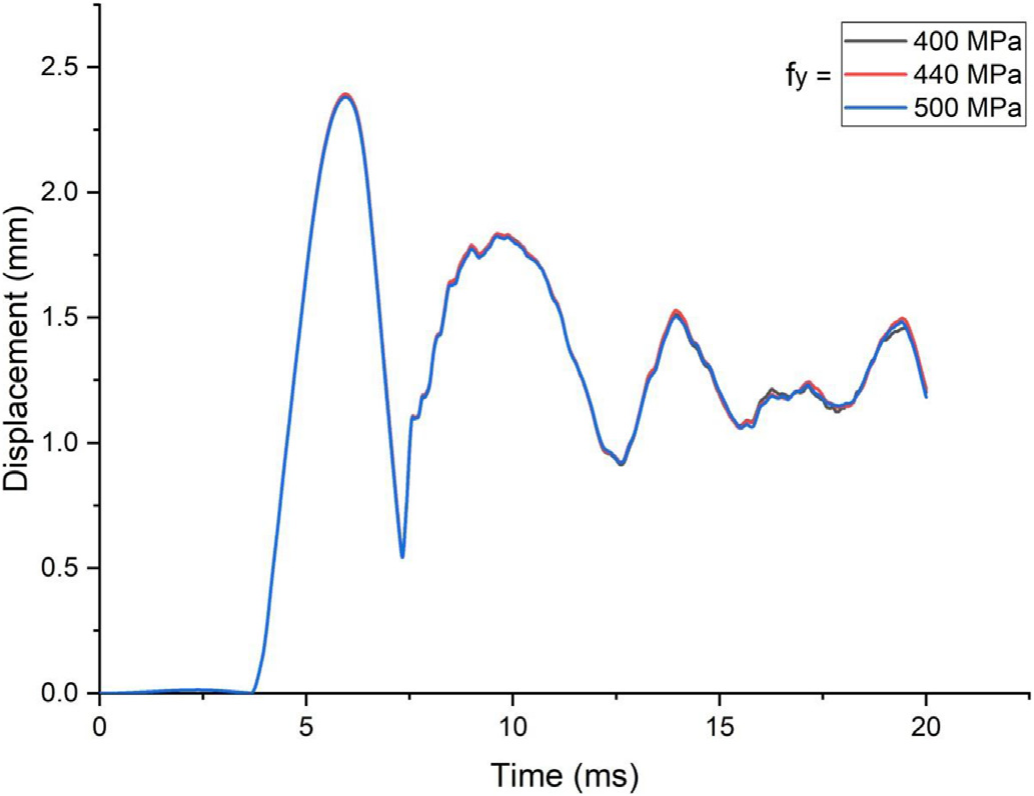
Displacement-time history plots for SCC beam with different yield stresses of reinforcement steel bars struck by 1.5 m/s impactor initial velocity.

**Figure 26:**
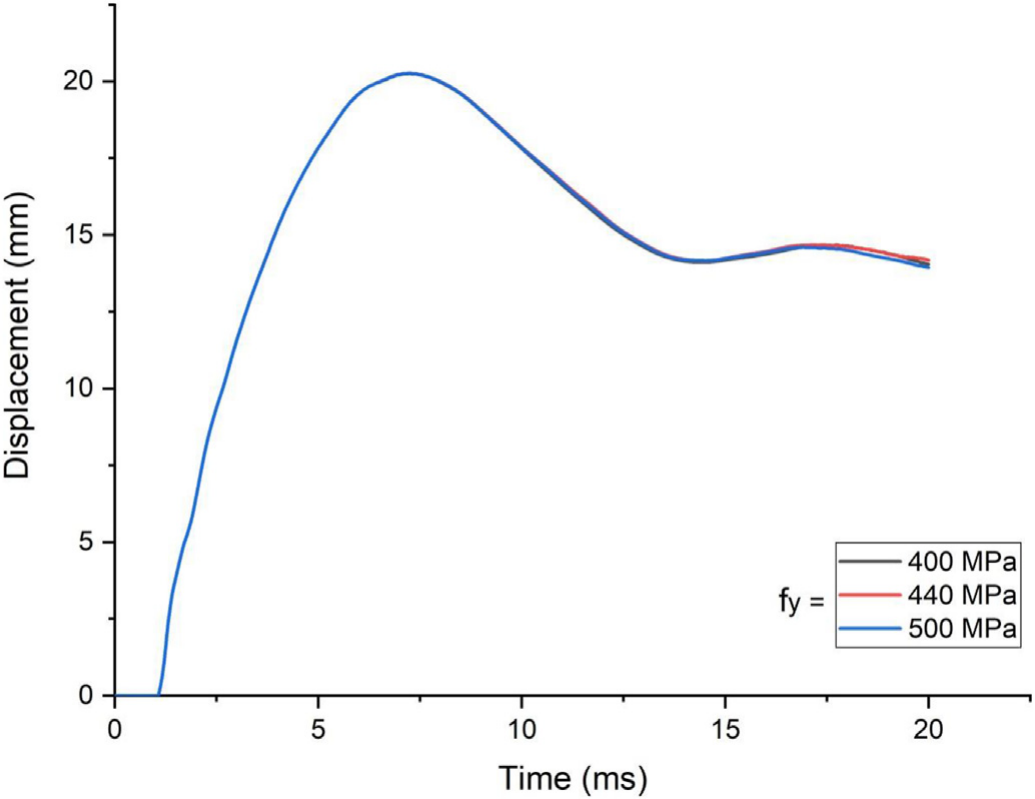
Displacement-time history plots for SCC beam with different yield stresses of reinforcement steel bars struck by 10 m/s impactor initial velocity.

**Figure 27:**
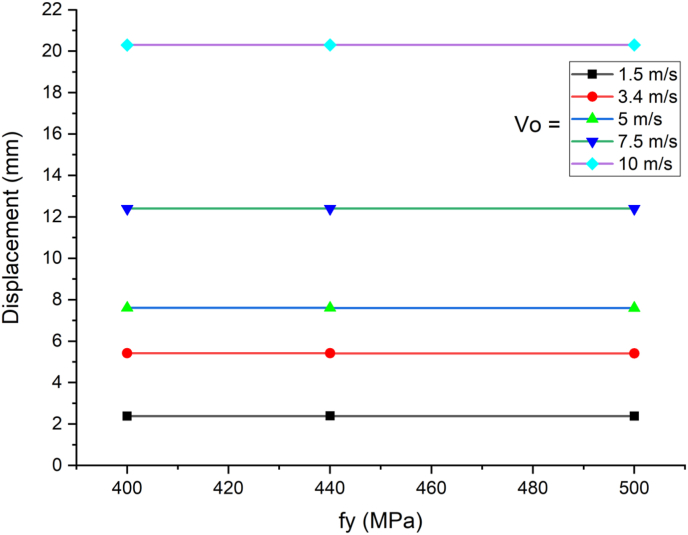
Comparison of displacement versus yield stresses of reinforcement steel bars (f_y_) for SCC beam under various ranges of impactor initial velocities (V_o_).

**Figure 28:**
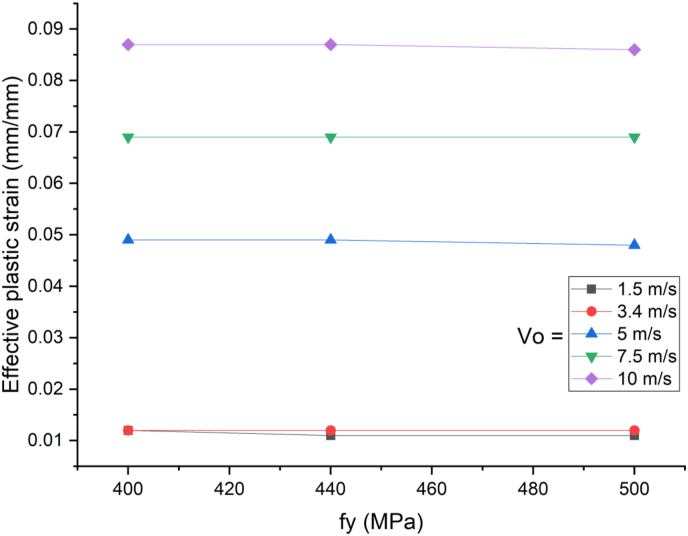
Comparison of effective plastic strain versus yield stress of reinforcement steel bars (f_y_) for SCC beam under various ranges of impactor initial velocities (V_o_).

This section presents dynamic response results of a SCC beam under combined blast-impact loading taking into consideration various yield stresses of reinforcement steel bar. Blast load of 12.5 kg charge mass and scaled distance Z = 1.72 $\left(\frac{m}{kg^{\frac{1}{3}}}\right)$ was applied into a SCC beam. As shown in Figures 25 and 26, varying rebar yield stress exhibited insignificant effect in improving deflection response quantity both in cases of blast load combined with low and high impacting initial velocity. Similar trend is also observed in peak deflection response quantity (Figure 27). The same is true in cases of effective plastic strain values (Figure 28).

### 3.12 Effect of varying flange thickness of H-type structural steel beam

**Figure 29:**
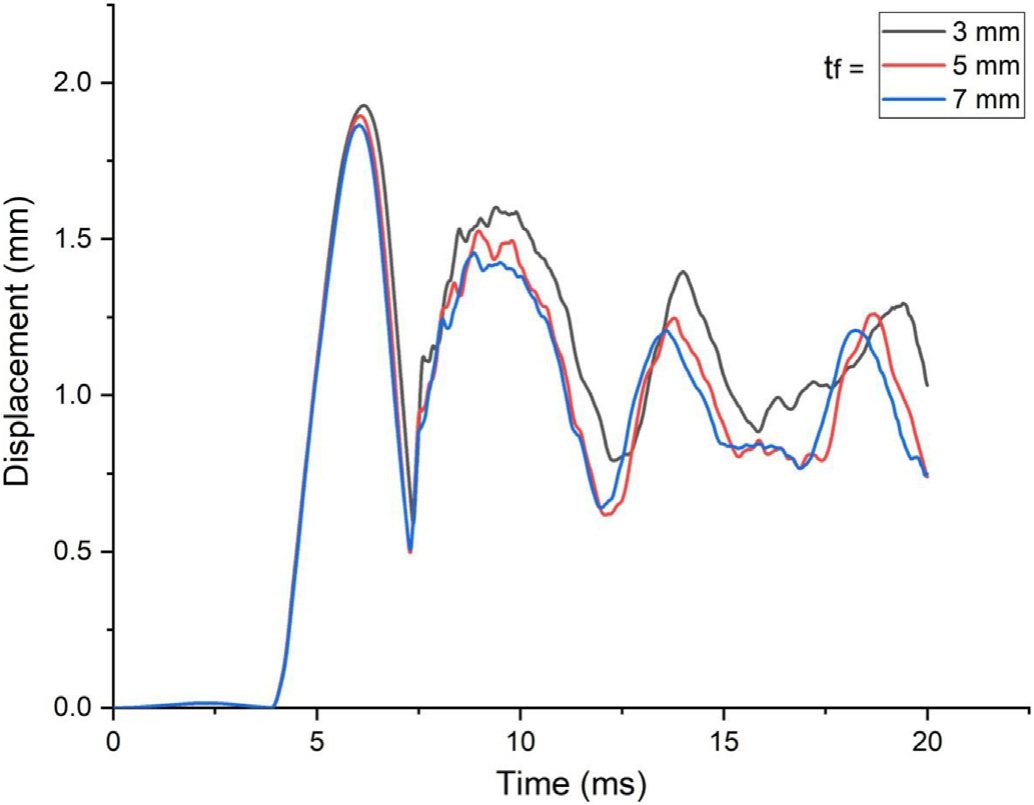
Displacement-time history plots for SCC beam with different flange thicknesses of H-type structural steel beam struck by 1.5 m/s impactor initial velocity.

**Figure 30:**
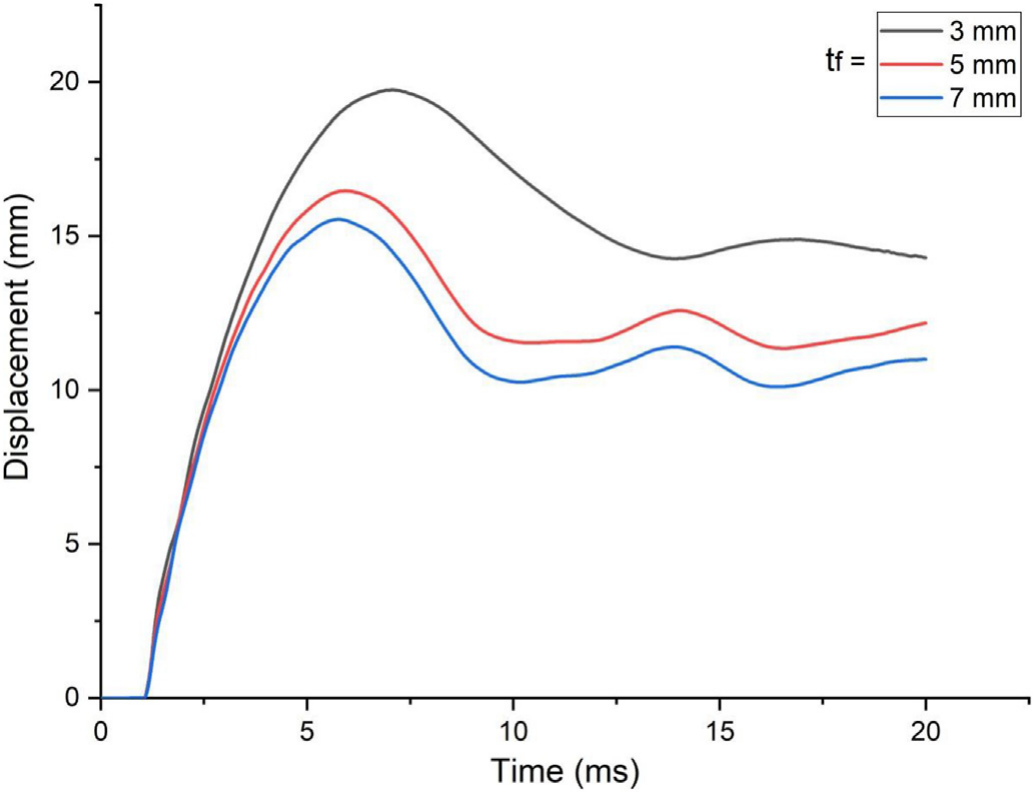
Displacement-time history plots for SCC beam with different flange thicknesses of H-type structural steel beam struck by 10 m/s impactor initial velocity.

**Figure 31:**
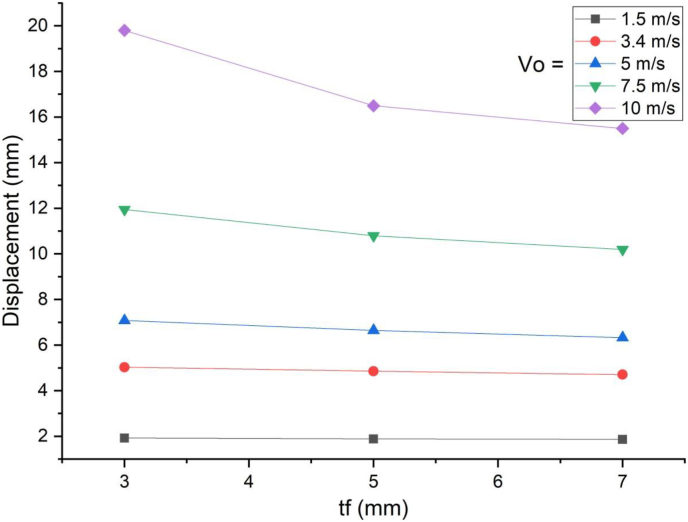
Comparison of displacement versus flange thicknesses of H-type structural steel beam (t_f_) for SCC beam under various ranges of impactor initial velocities (V_o_).

**Figure 32:**
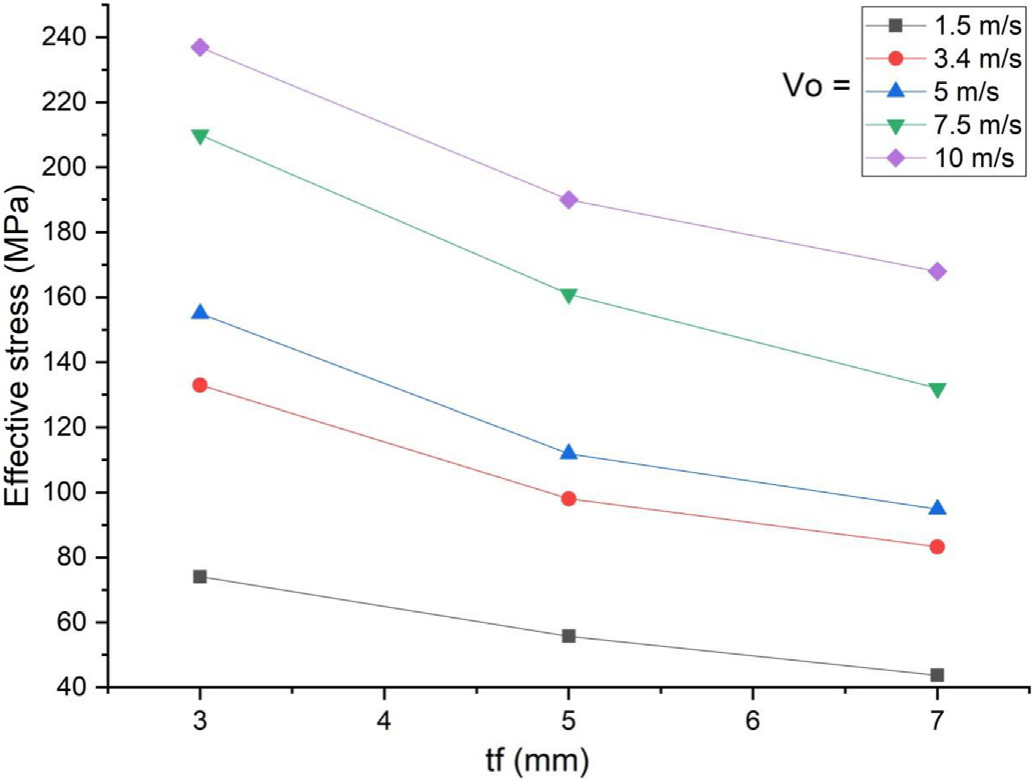
Comparison of effective stress versus flange thickness of H-type structural steel beam (t_f_) for SCC beam under various ranges of impactor initial velocities (V_o_)

This section presents parametric finite element analysis results of employing various types of flange thicknesses (3 mm, 5 mm and 7 mm) of H-type structural steel beam in a SCC beam. Blast load of 10 kg charge mass and scaled distance Z = 1.86 $\left(\frac{m}{kg^{\frac{1}{3}}}\right)$ was applied on a SCC beam. In cases of blast load combined with low impacting velocity, comparing flange thicknesses of H-type structural steel beam with 3 mm with 5 mm and 7 mm revealed minor difference in peak nodal displacement value (Figure 29) however this trend changes when a SCC beam subjected to blast load combined with high impacting speed. As the impacting speed was raised to 10 m/s, varying flange thicknesses of H-type structural steel beam from 3 mm to 5 mm and 7 mm showed significant 17% and 22% decrease in peak displacement respectively (Figure 30). Moreover, in post peak behavior of load deflection plot, similar improvement in deflection response quantity was observed. Figures 31 and 32 also shows improved performance gains in terms of peak nodal displacement and effective plastic strain as flange thickness increases from 3 mm to 5 mm and 7 mm. This performance gain is more visible specially for blast load combined with high velocity impact loading.

### 3.13 Effect of varying web thickness

**Figure 33:**
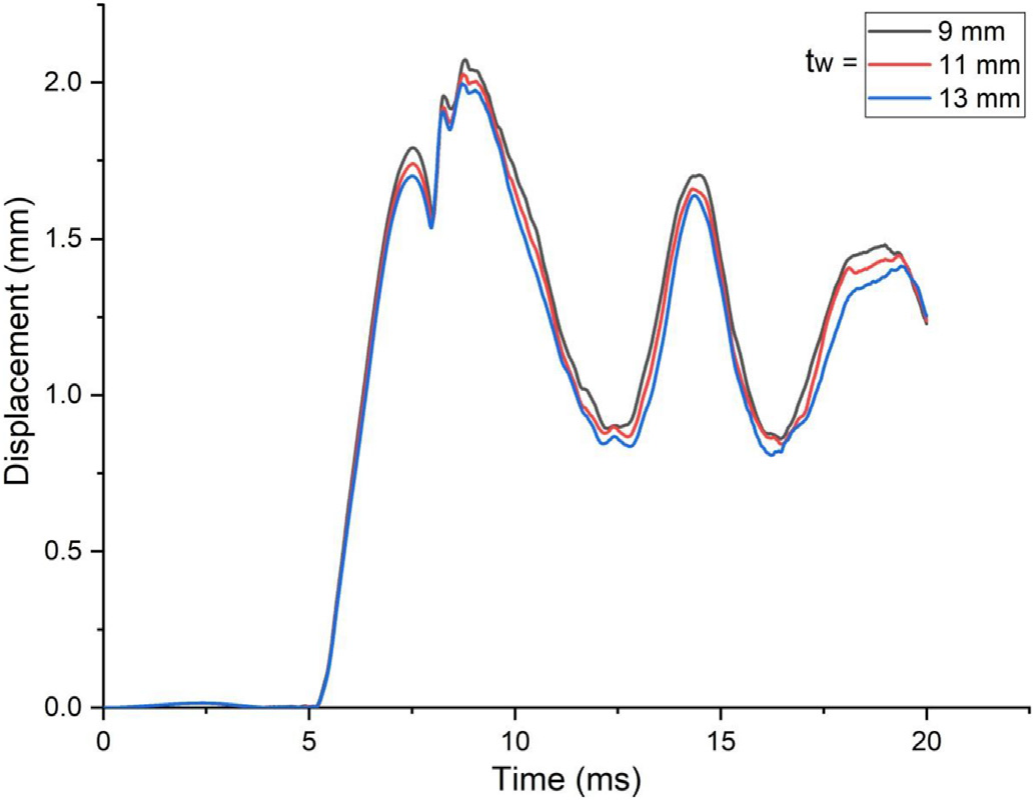
Displacement-time history curves for SCC beam with different web thicknesses of H-type structural steel beam struck by 1.5 m/s impactor initial velocity.

**Figure 34:**
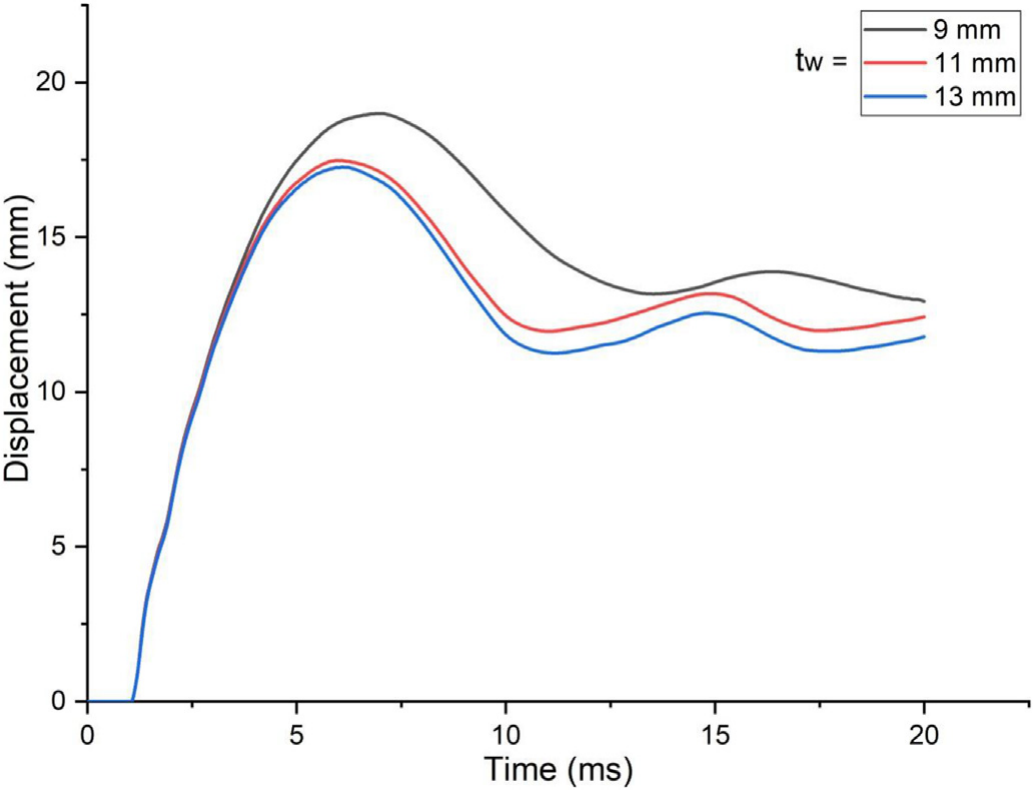
Displacement-time history curves for SCC beam with different web thicknesses of H-type structural steel beam struck by 10 m/s impactor initial velocity.

**Figure 35:**
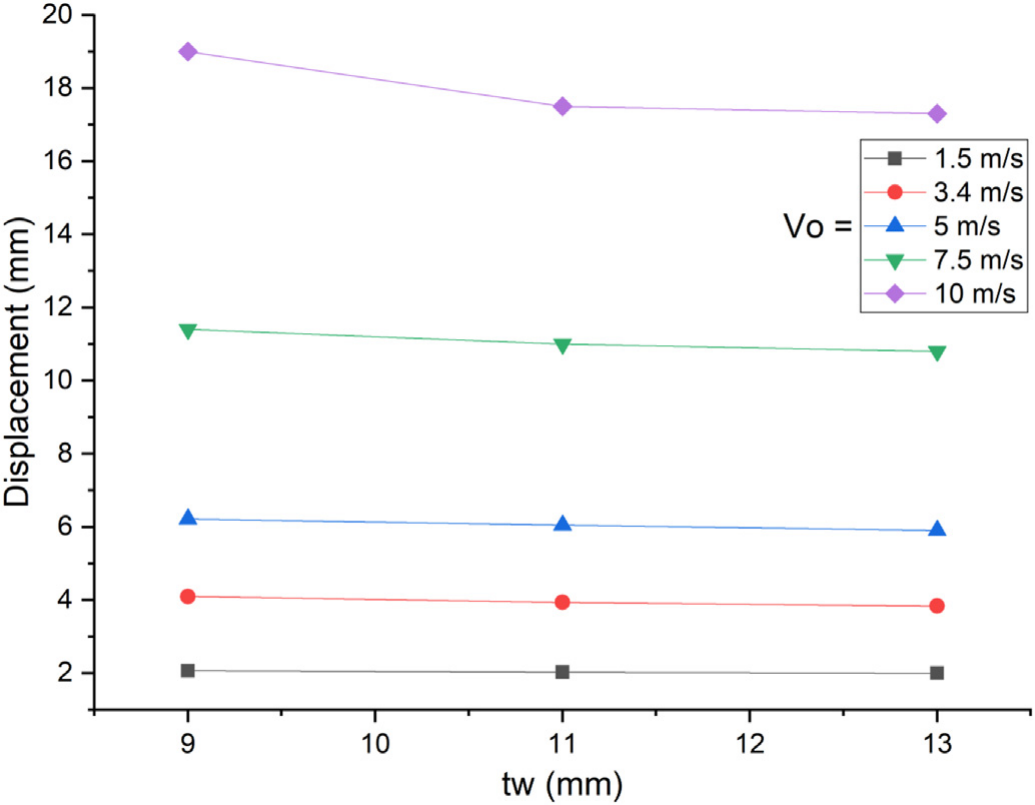
Comparison of displacement versus web thicknesses of H-type structural steel beam (t_w_) for SCC beam under various ranges of impactor initial velocities (V_o_).

**Figure 36:**
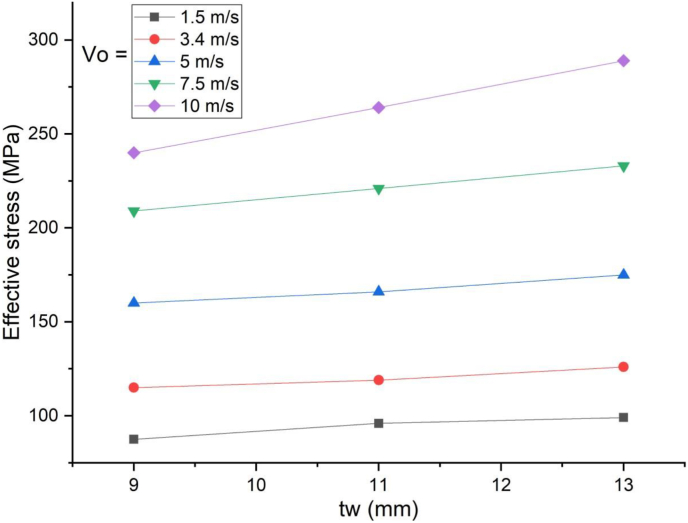
Comparison of effective stress versus web thickness of H-type structural steel beam (t_w_) for SCC beam under various ranges of impactor initial velocities (V_o_)

The response of a SCC beam under combined blast-impact load with different web thicknesses (9 mm, 11 mm and 13 mm) of H-type structural steel beam was studied by taking into account an enhanced blast load with 15 kg charge mass and scaled distance Z = 2.03$\left(\frac{m}{kg^{\frac{1}{3}}}\right)$. When a SCC beam subjected to combined blast and low impacting initial velocities, negligible difference in nodal displacement values were observed (Figure 33) however this trend changed when applied impacting energy raised by increasing impacting speed and for a blast scenario with 10 m/s impactor initial velocity, as compared to 9 mm web thicknesses of H-type structural steel beam, 11 mm and 13 mm web thickness showed 8% and 9% decrease in peak displacement, respectively (Figure 34). Also, nodal displacement values were reduced and effective stress values were peaked as web of H-type structural steel beam increased from 9 mm to 11 mm (Figures 35 and 36).

## 4 Conclusions

This study presents numerical investigation on response of steel concrete composite (SCC) beam subjected to combined blast-impact loads. Experimental data reported in literature was used to validate and verify accuracy and reliability developed finite element models. Further, detailed parametric studies were performed on validated FE model on various influential variables such as concrete grade, stud and on embedded structural steel characteristics to get insight into behavior of steel concrete composite (SCC) beam under combined blast-impact loads. Combined effect of blast-impact loading was simulated in FE model by using enhanced blast loading, extracted from CONWEP manual and a box-like drop weight with prescribed initial velocity. Nodal displacement time history and peak values, effective stress, and effective plastic strain value outputs were extracted and discussed in detail to describe and evaluate dynamic behavior a SCC beam under combined blast – impact loading. Next, major findings of the research are presented.

• Varying material strengths specifically compressive strength of concrete and yield stress of H-type structural steel beam have effect on response of a SCC beam under combined blast-impact load. Their effect is significant as speed of impactor increases. Also, as material strength increases with larger impactor velocity, effect of strain rate became more viable.
• Use of high strength studs and reinforcement steel bars has negligible influence in improving displacement response quantity of a SCC beam under combined blast-impact loading.
• Increasing flange and web thickness of H-type structural steel beam significantly improves displacement response quantity of a SCC beam under combined blast-impact loading. This performance gain materializes more as impactor initial velocity increases.
• Raising impactor initial velocities enormously affects dynamic response behavior of a SCC beam including nodal displacement and developed stresses values.
• Overall, SCC beam failed due to excess concrete damage under the action of combined blast - impact loading.

## Declarations

### Author contribution statement

Tesfaye Alemu Mohammed (PhD); Solomon Abebe: Conceived and designed the experiments; Performed the experiments; Analyzed and interpreted the data; Contributed reagents, materials, analysis tools or data; Wrote the paper.

### Funding statement

This research did not receive any specific grant from funding agencies in the public, commercial, or not-for-profit sectors.

### Data availability statement

Data included in article/supp. material/referenced in article.

### Declaration of interest’s statement

The authors declare no conflict of interest.

### Additional information

No additional information is available for this paper.
